# Water quality as an economic barrier to photocatalytic green hydrogen production

**DOI:** 10.1039/d6ra06815d

**Published:** 2026-09-07

**Authors:** Afsar Khan, Munevver Tuna Genc, Imren Hatay Patir, Mustafa Ersoz

**Affiliations:** a Department of Chemistry, Selçuk University 42030 Konya Türkiye mersoz@selcuk.edu.tr; b Department of Biotechnology, Selçuk University 42030 Konya Türkiye imrenhatay@selcuk.edu.tr

## Abstract

Photocatalytic water splitting has emerged as a promising technology for sustainable green hydrogen production because it directly converts solar energy into chemical energy under mild operating conditions. Although extensive research has focused on developing highly efficient photocatalysts, cocatalysts, and charge-transfer strategies, the influence of water quality on photocatalytic hydrogen production remains comparatively underexplored. In practical applications, hydrogen generation is expected to utilize a wide range of water resources, including freshwater, groundwater, seawater, municipal wastewater, industrial effluents, and reclaimed water, all of which contain dissolved inorganic ions, natural organic matter, microorganisms, and suspended solids that can adversely affect photocatalytic performance. These contaminants may adsorb onto catalyst surfaces, block active sites, alter surface chemistry, promote charge-carrier recombination, induce photocorrosion, and reduce long-term catalyst stability, ultimately decreasing hydrogen production efficiency. Furthermore, maintaining the high water quality required for stable operation often necessitates energy-intensive and costly pretreatment processes, making water purification an important but frequently overlooked contributor to the overall cost of green hydrogen production. This review critically examines the relationship between water quality and photocatalytic hydrogen evolution by discussing the effects of major inorganic ions, heavy metals, natural organic matter, salinity, and real water matrices on catalyst activity and durability. Finally, current challenges, research gaps, and future strategies—including contaminant-tolerant photocatalysts, self-cleaning catalyst surfaces, integrated water treatment–hydrogen production systems, and standardized testing protocols using realistic water sources—are highlighted to facilitate the practical deployment of photocatalytic hydrogen production. By integrating photocatalysis, water chemistry, catalyst stability, and economic considerations, this review provides a comprehensive framework for advancing water-resilient and cost-effective solar hydrogen technologies.

## Abstract 2

Photocatalytic water splitting has emerged as a promising technology for sustainable green hydrogen production because it directly converts solar energy into chemical energy under mild operating conditions.

## Graphical abstract

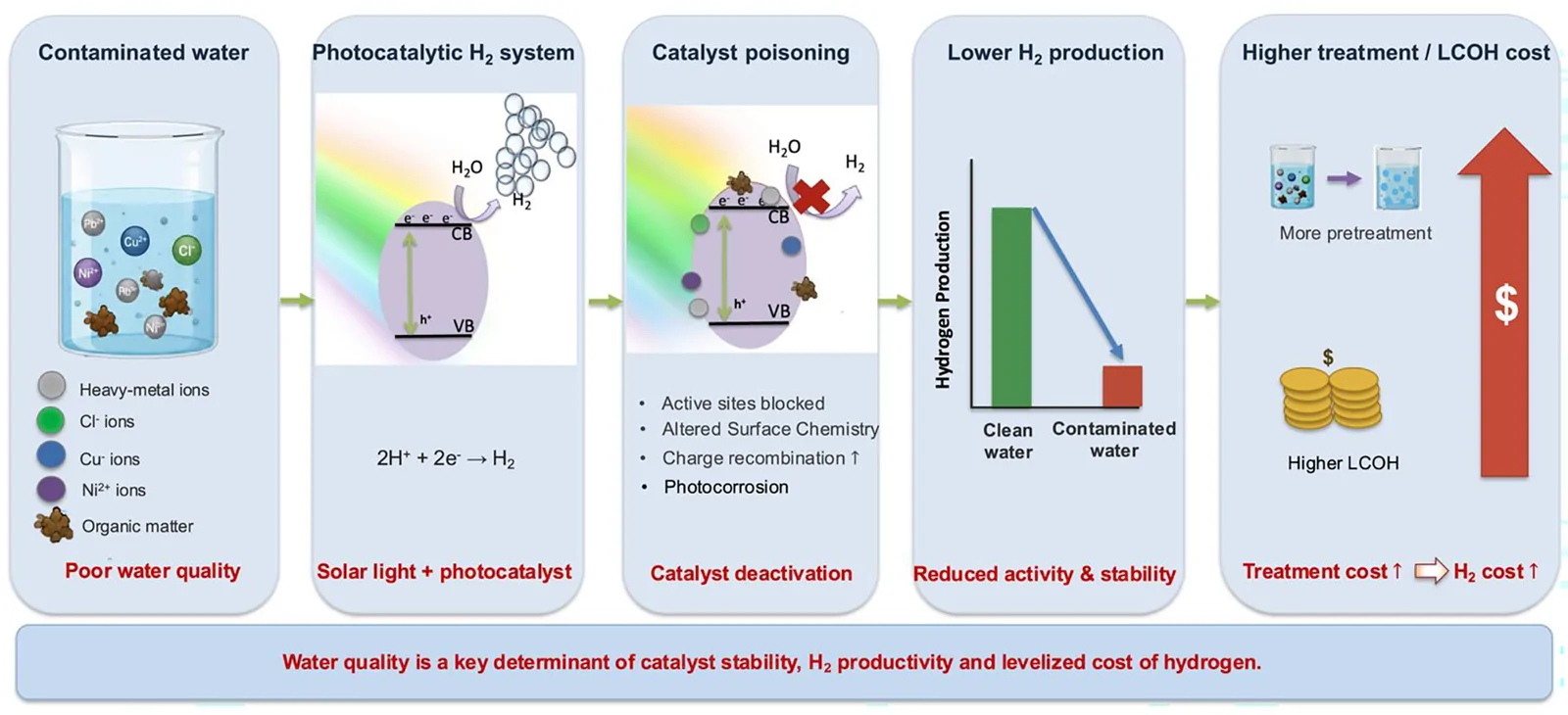

## 1. Introduction

Hydrogen has emerged as one of the most promising clean energy carriers for achieving global carbon neutrality because its utilization produces only water without direct carbon dioxide emissions.^1,2^ However, nearly 95% of the world's hydrogen is still produced from fossil fuels through processes such as steam methane reforming and coal gasification, resulting in substantial greenhouse gas emissions.^1^ To overcome this challenge, considerable attention has shifted toward renewable hydrogen production technologies powered by sustainable energy sources. Among these, photocatalytic water splitting is particularly attractive because it enables the direct conversion of solar energy into hydrogen under ambient conditions using semiconductor photocatalysts.^1,3,4^ Since the pioneering discovery of photoelectrochemical water splitting by Fujishima and Honda in 1972,^5^ significant progress has been achieved in the development of highly efficient photocatalysts through band-gap engineering, heterojunction construction, cocatalyst loading, defect engineering, and nanostructure design. Despite these advances, the practical application of photocatalytic hydrogen production remains limited by several challenges, including low solar-to-hydrogen conversion efficiency, catalyst stability, and the influence of real water chemistry.^4,6,7^

Most studies on photocatalytic hydrogen production have been conducted using ultrapure or deionized water under carefully controlled laboratory conditions.^8–10^ In contrast, large-scale hydrogen production is unlikely to rely exclusively on high-purity water because freshwater resources are increasingly constrained by population growth, industrialization, climate change, and competing agricultural demands.^9,11^ Future hydrogen production facilities are therefore expected to utilize alternative water sources, including groundwater, seawater, municipal wastewater, industrial effluents, and reclaimed water. These water sources contain a wide range of dissolved inorganic ions, heavy metals, natural organic matter, microorganisms, and suspended particles that can significantly influence photocatalytic performance.^12,13^ Depending on their concentration and chemical nature, these contaminants may adsorb onto catalyst surfaces, block active sites, alter surface electronic properties, promote charge-carrier recombination, induce photocorrosion, or compete with the hydrogen evolution reaction, ultimately decreasing hydrogen production efficiency and catalyst lifetime. In some cases, certain ions may even enhance photocatalytic activity by acting as electron scavengers or modifying surface reaction pathways, highlighting the complexity of contaminant–photocatalyst interactions^12–14^

Beyond their influence on catalyst performance, water contaminants also have important economic implications. Theoretical water consumption for producing 1 kg of hydrogen is approximately 9 L; however, practical hydrogen production systems require significantly more water because of purification losses, cooling, membrane flushing, and other auxiliary processes.^8,14,15^ Consequently, maintaining the water quality required for efficient hydrogen production often necessitates pretreatment technologies such as filtration, softening, desalination, deionization, or advanced oxidation processes, all of which increase energy consumption, capital investment, and operating costs. Although electricity demand and photocatalyst development have received considerable attention in techno-economic analyses of green hydrogen production, the contribution of water quality and water treatment to the overall hydrogen production cost has remained comparatively underexplored. This knowledge gap is particularly important for regions facing freshwater scarcity, where access to high-quality water may become a limiting factor for the large-scale deployment of photocatalytic hydrogen technologies.^10,16^

This review critically examines the role of water quality in photocatalytic green hydrogen production by integrating recent advances in photocatalysis, water chemistry, catalyst poisoning, and techno-economic analysis. The review first discusses water consumption and water quality requirements in different hydrogen production technologies before summarizing the effects of major inorganic ions, heavy metals, natural organic matter, salinity, and real water matrices on photocatalytic hydrogen evolution. It further highlights the mechanisms of catalyst poisoning and deactivation, the experimental and computational techniques used to investigate contaminant catalyst interactions, and recent strategies for developing contaminant-tolerant photocatalysts and integrated water treatment–hydrogen production systems. Finally, future research priorities are identified to facilitate the development of water-resilient, economically viable, and scalable photocatalytic hydrogen production technologies for sustainable energy applications.

## 2. Hydrogen economy

The transition toward a hydrogen economy has emerged as a key strategy for achieving global carbon neutrality and enhancing energy security by replacing fossil fuels with low-carbon energy carriers.^17–19^ Hydrogen possesses a high gravimetric energy density (120 MJ kg^−1^), nearly three times that of gasoline, and produces only water as a by-product when utilized in fuel cells, making it an attractive clean energy vector for sectors that are difficult to electrify, including steel production, chemical manufacturing, heavy transportation, shipping, and aviation.^17^ However, the environmental benefits of hydrogen depend strongly on its production pathway.^19^ At present, the vast majority of global hydrogen is produced from fossil fuels through steam methane reforming and coal gasification, resulting in substantial greenhouse gas emissions. According to the International Energy Agency (IEA) (Table 1), global hydrogen demand reached approximately 97 million tonnes (Mt) in 2023 and is expected to approach 100 Mt in 2024, yet low-emission hydrogen accounts for less than 1% of total production.^19^ The IEA further projects that announced low-emission hydrogen projects could reach approximately 49 Mt per year by 2030, although only a fraction has reached final investment decisions, highlighting the significant technological and economic barriers that remain. Consequently, considerable research efforts are focused on sustainable hydrogen production technologies, including water electrolysis, photoelectrochemical water splitting, and photocatalytic water splitting, which can directly convert solar energy into hydrogen without carbon emissions. Among these approaches, photocatalytic water splitting has attracted increasing attention because of its potential for low-cost, decentralized hydrogen production using abundant solar energy and water. Nevertheless, its large-scale commercialization is still hindered by low solar-to-hydrogen efficiency, photocatalyst instability, and, importantly, the adverse effects of water quality and catalyst poisoning, which can significantly reduce hydrogen productivity and increase production costs. Therefore, understanding the relationship between water quality, catalyst durability, and economic feasibility is becoming increasingly important for realizing a sustainable hydrogen economy.^1,2,17,19^

**Table 1:** Key indicators of the hydrogen economy

Indicator	Quantitative value	Significance	Reference
Hydrogen energy density	120 MJ kg^−1^	∼3× higher than gasoline (gravimetric)	17
Global hydrogen demand (2023)	≈97 Mt	Growing industrial demand	20
Projected demand (2024)	≈100 Mt	Continued market growth	
Low-emission hydrogen share	<1%	Most H_2_ still fossil-fuel based	21
Announced low-emission capacity by 2030	≈49 Mt per year	Depends on project completion	21
Major production routes	SMR, coal gasification, electrolysis, PEC, photocatalysis	Determine emissions and cost	17 and 22
Key commercialization barriers	High cost, low efficiency, catalyst instability, water quality	Limits commercialization	22

### 2.1. Photocatalytic water splitting

Photocatalytic water splitting has emerged as one of the most promising technologies for sustainable hydrogen production because it directly converts abundant solar energy into chemical energy without emitting greenhouse gases.^17,23,24^ Unlike conventional hydrogen production methods that rely on fossil fuels, photocatalytic water splitting utilizes semiconductor photocatalysts to drive the decomposition of water into hydrogen (H_2_) and oxygen (O_2_) under light irradiation.^9,25^ Since the pioneering work of Fujishima and Honda,^5^ which demonstrated photoelectrochemical water splitting on TiO_2_ electrodes, extensive research has focused on developing efficient photocatalysts capable of utilizing visible sunlight while maintaining high stability and low production costs. The principal advantage of photocatalysis is its ability to integrate solar energy harvesting and catalytic hydrogen evolution into a single-step process without requiring external electrical energy, making it particularly attractive for decentralized and off-grid hydrogen production systems. Consequently, photocatalytic hydrogen generation has become an important component of future renewable energy strategies aimed at reducing dependence on fossil fuels and mitigating climate change.^10,11,22,26^

From an environmental perspective, photocatalytic water splitting offers several advantages over conventional hydrogen production technologies. Currently, approximately 95% of global hydrogen production originates from steam methane reforming, coal gasification, and other fossil fuel-based processes that release significant quantities of carbon dioxide into the atmosphere. In contrast, photocatalytic water splitting consumes only sunlight and water, producing hydrogen with virtually zero direct carbon emissions. Moreover, photocatalysts can operate under ambient temperature and pressure, eliminating the high-temperature reactors and large energy inputs required by thermochemical processes. When coupled with renewable water resources or treated wastewater, photocatalytic systems can contribute simultaneously to clean energy generation and water remediation, thereby supporting multiple Sustainable Development Goals (SDGs), including affordable clean energy, clean water, and climate action. Hisatomi *et al.*^10^ emphasized that particulate photocatalysts are particularly attractive because they can be scaled into large-area solar reactors with relatively simple engineering compared with photoelectrochemical cells.

Economically, photocatalytic water splitting has the potential to reduce hydrogen production costs by eliminating electricity consumption, which represents the largest operating expense in conventional water electrolysis.^11^ Electrolytic hydrogen production requires a continuous electrical input and highly purified water, whereas photocatalytic systems utilize solar photons as the primary energy source.^22^ Although present photocatalytic efficiencies remain lower than those of commercial electrolyzers, advances in semiconductor engineering, cocatalyst modification, heterojunction construction, defect engineering, and single-atom catalysis have significantly improved hydrogen evolution rates during the past decade.^22^ Nevertheless, several challenges continue to limit commercialization, including low solar-to-hydrogen conversion efficiency, rapid charge carrier recombination, photocorrosion, catalyst instability, and sensitivity to water contaminants. These challenges become particularly important when photocatalysts are exposed to real water sources containing dissolved metal ions, inorganic salts, natural organic matter, and suspended solids that poison active sites and reduce hydrogen production efficiency. Therefore, improving catalyst durability under realistic water conditions has become a critical research direction for achieving economically viable photocatalytic hydrogen production.^27^

### 2.2. Mechanism of photocatalytic water splitting

The photocatalytic water splitting process consists of several sequential physicochemical steps that occur on the semiconductor surface under light irradiation (Fig. 1). Initially, when photons with energy equal to or greater than the semiconductor band gap strike the photocatalyst, electrons in the valence band (VB) are excited to the conduction band (CB), leaving positively charged holes in the valence band:Photocatalyst + *ℏν* → e_CB_^−^ + h_VB_^+^

**Fig. 1:**
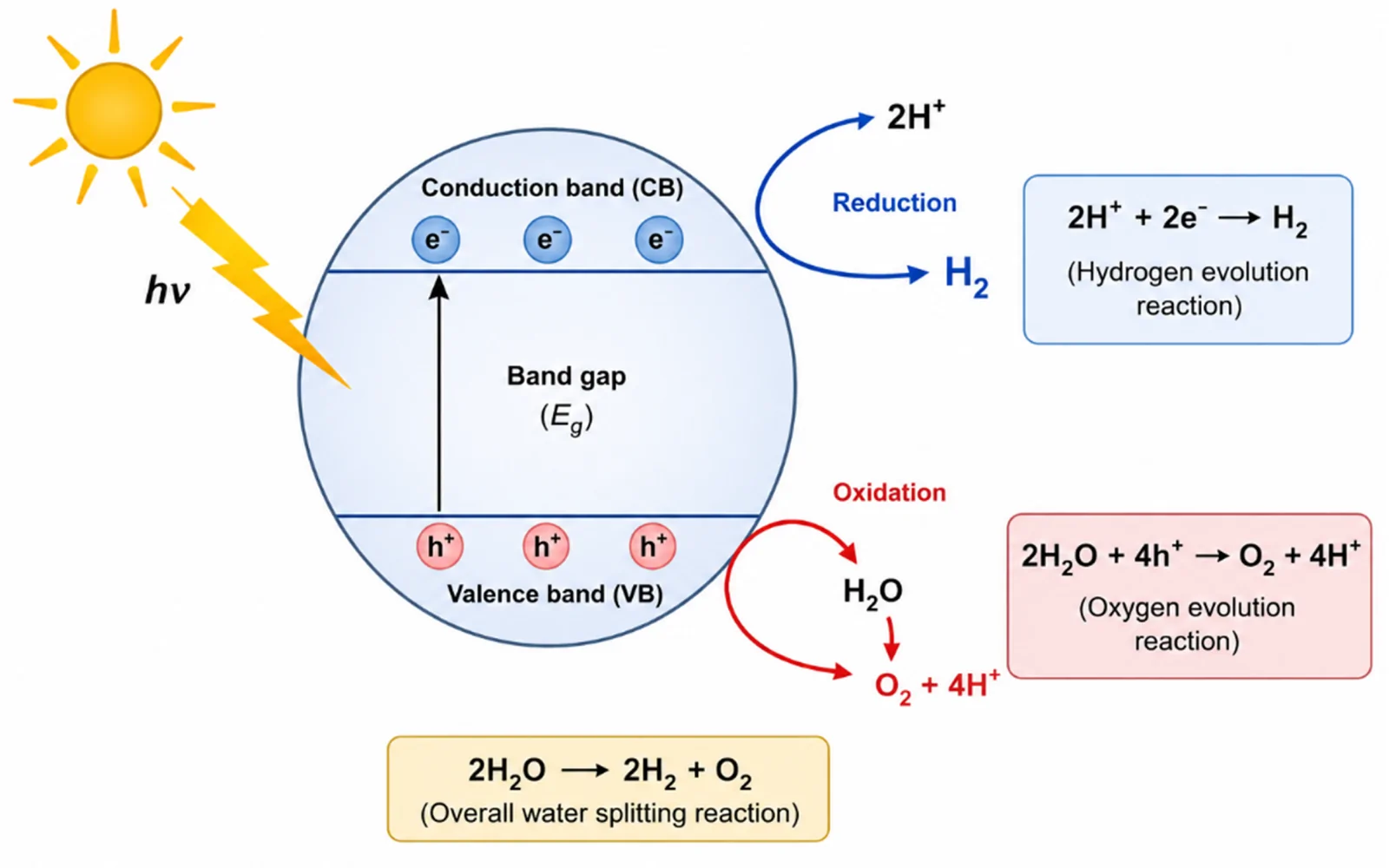
Schematic illustration of the mechanism of photocatalytic water splitting on a semiconductor photocatalyst.^22^

The photogenerated electrons and holes subsequently migrate toward the catalyst surface. Efficient charge separation is essential because rapid electron–hole recombination converts absorbed solar energy into heat rather than useful chemical energy. Surface defects, cocatalysts, heterojunctions, and internal electric fields are commonly introduced to enhance charge separation and prolong carrier lifetime.

At the reduction sites, conduction-band electrons reduce protons to produce molecular hydrogen according to:2H^+^ + 2e^−^ → H_2_

Simultaneously, valence-band holes oxidize water molecules to generate oxygen:2H_2_O + 4h^+^ → O_2_ + 4H^+^

The overall water splitting reaction is therefore:2H_2_O → 2H_2_ + O_2_with a standard Gibbs free energy change of approximately 237 kJ mol^−1^, corresponding to a minimum thermodynamic potential of 1.23 V. To accomplish overall water splitting, the conduction band minimum of the photocatalyst must be more negative than the H^+^/H_2_ reduction potential (0 V *vs.* NHE), while the valence band maximum must be more positive than the H_2_O/O_2_ oxidation potential (+1.23 V *vs.* NHE). Consequently, both appropriate band-edge alignment and efficient charge separation are indispensable for high photocatalytic activity.

In practical systems, cocatalysts such as Pt, Rh, Ni, Co, and MoS_2_ are frequently deposited on semiconductor surfaces to accelerate proton reduction and suppress charge recombination. Likewise, sacrificial electron donors (*e.g.*, methanol, triethanolamine, or eosin Y/TEOA systems in laboratory studies) are often employed to consume photogenerated holes and improve apparent hydrogen evolution rates during catalyst evaluation. However, for sustainable green hydrogen production, the ultimate objective remains overall water splitting using pure water without sacrificial reagents. Under real operating conditions, dissolved contaminants including heavy metal ions, inorganic anions, natural organic matter, and suspended particles may adsorb on active sites, block reaction centers, modify surface electronic states, and increase charge recombination, thereby decreasing hydrogen production efficiency. These poisoning phenomena represent one of the major barriers to the practical deployment of photocatalytic water splitting technologies and underscore the importance of understanding water quality effects in the design of next-generation contaminant-tolerant photocatalysts.^7,12,28^

## 3. Water requirements for photocatalytic hydrogen production

### 3.1. Water consumption in different hydrogen technologies

Water is the sole reactant in photocatalytic water splitting, where solar energy is directly converted into chemical energy through semiconductor photocatalysts to produce hydrogen and oxygen.^26^ The overall reaction can be expressed as:2H_2_O → 2H_2_ + O_2_

Based on this reaction, the theoretical stoichiometric requirement is 9 kg (approximately 9 L) of water to produce 1 kg of hydrogen. This value represents only the water molecules consumed during the hydrogen evolution reaction and assumes complete conversion without any losses. In practice, however, the actual water demand is considerably higher because water is also required for catalyst washing, reactor cleaning, cooling, evaporation compensation, sampling, and other auxiliary operations. Although photocatalytic water splitting is still predominantly at the laboratory and pilot scales, these additional water requirements are expected to become increasingly important as the technology moves toward commercial deployment. Consequently, practical water consumption will depend not only on the reaction stoichiometry but also on reactor configuration, catalyst stability, water recycling efficiency, and operating conditions.^3^

Unlike electrolysis, photocatalytic hydrogen production is highly sensitive to the composition of the reaction medium because the photocatalyst is in direct contact with water throughout the reaction.^28^ Most laboratory studies therefore employ ultrapure or deionized water to eliminate the influence of dissolved ions, heavy metals, natural organic matter, and suspended solids.^28^

However, the use of high-purity water increases operational costs and limits the sustainability of large-scale hydrogen production, particularly in water-stressed regions. Future commercial systems are expected to utilize alternative water resources, including groundwater, surface water, seawater, municipal wastewater, and reclaimed water.^20^ These water sources contain various contaminants that can influence photocatalytic reactions by modifying surface chemistry, blocking active sites, promoting charge-carrier recombination, or accelerating catalyst deactivation.^29^ Consequently, the actual water requirement for photocatalytic hydrogen production should be considered not only in terms of quantity but also in terms of water quality.^20^ From an economic perspective, water consumption in photocatalytic hydrogen production extends beyond the volume directly consumed in the reaction. Additional water and energy are often required for pretreatment processes such as filtration, softening, deionization, desalination, and the removal of natural organic matter to maintain catalyst performance and ensure long-term operational stability.^29–31^ These pretreatment steps contribute to both capital and operating costs and may significantly influence the overall cost of green hydrogen production.^21^ Despite substantial advances in photocatalyst development, relatively few studies have evaluated the economic impact of feedwater quality or quantified the trade-off between water purification costs and photocatalytic efficiency.^28^ As hydrogen production scales up, integrating contaminant-tolerant photocatalysts with cost-effective water treatment technologies will be essential for reducing water consumption and improving the sustainability of photocatalytic hydrogen production.^30^

### 3.2. Water quality as a neglected challenge

Water quality has long been recognized as a critical requirement for conventional electrolysis; however, its influence on photocatalytic water splitting has received comparatively little attention despite the direct and continuous contact between the photocatalyst and the reaction medium.^23,29,32,33^ Most laboratory investigations evaluate photocatalytic performance using ultrapure or deionized water to eliminate interference from dissolved ions, heavy metals, natural organic matter, microorganisms, and suspended solids. Although this approach enables accurate assessment of intrinsic photocatalytic activity, it does not represent the chemical complexity of natural waters or industrial wastewaters that would be encountered in practical applications. As Hisatomi *et al.*^11^ emphasized, translating photocatalytic water splitting from laboratory research to industrial deployment requires consideration of realistic operating environments, where the chemical composition of water can strongly influence catalyst stability, charge-transfer kinetics, and hydrogen evolution performance. Similarly, Nishioka *et al.*^1^ noted that the long-term durability of photocatalytic systems depends not only on the intrinsic properties of semiconductor materials but also on the quality of the reaction medium, which has often been overlooked in catalyst design and performance evaluation.

The presence of dissolved contaminants introduces multiple challenges that extend beyond simple reductions in hydrogen production. Heavy metal ions such as Pb^2+^, Ni^2+^, Cu^2+^, and Hg^2+^ can adsorb strongly onto catalyst surfaces, blocking active reaction sites and altering the electronic structure of cocatalysts. Meanwhile, inorganic anions including phosphate, carbonate, sulfate, chloride, and nitrate may compete with water molecules for adsorption sites or act as scavengers of photogenerated charge carriers, thereby reducing photocatalytic efficiency.^16,23,29^ In addition, natural organic matter, particularly humic and fulvic substances, can form surface coatings that inhibit light absorption and impede mass transfer between the catalyst and water. Chong *et al.*^24^ demonstrated that natural water constituents significantly influence photocatalytic reaction pathways by competing for reactive oxygen species and occupying catalytically active sites, while Kudo and Miseki^27^ highlighted that surface poisoning and rapid charge-carrier recombination remain among the major limitations preventing efficient overall water splitting.

Another reason why water quality has remained underexplored is that the primary focus of photocatalysis research has historically been directed toward developing new semiconductor materials with improved light absorption, narrower band gaps, heterojunction architectures, cocatalyst loading, and defect engineering. Considerable advances have been achieved in catalyst synthesis; however, comparatively few studies have systematically evaluated catalyst performance in realistic water matrices. As Maeda and Domen^34^ pointed out, improving photocatalytic activity alone is insufficient for practical implementation unless catalyst stability under real operating conditions is simultaneously addressed. More recently, Kranz and Wächtler^35^ argued that advanced characterization techniques capable of monitoring catalyst surfaces under realistic reaction environments are essential for understanding degradation mechanisms and designing more robust photocatalytic materials.

From an engineering and economic perspective, neglecting water quality may lead to significant overestimation of photocatalytic performance and underestimation of production costs. Catalysts evaluated exclusively in ultrapure water often exhibit substantially lower hydrogen evolution rates when exposed to river water, groundwater, seawater, or wastewater because contaminant adsorption accelerates catalyst deactivation and shortens catalyst lifetime. Consequently, additional pretreatment processes—including filtration, softening, reverse osmosis, ion exchange, and activated carbon adsorption—may become necessary to maintain stable operation, increasing both capital and operating costs. Maurya *et al.*^36^ demonstrated through techno-economic analysis that catalyst durability and operating efficiency are among the dominant parameters controlling the levelized cost of hydrogen (LCOH), while recent assessments by the International Energy Agency (IEA) emphasize that water availability and quality are becoming increasingly important considerations as hydrogen production expands globally. Therefore, integrating water quality considerations into photocatalyst design, reactor engineering, and techno-economic assessments represents a critical research priority for the commercialization of photocatalytic hydrogen production. Rather than treating water solely as a reactant, future research should consider it as a dynamic chemical environment capable of governing catalyst stability, reaction kinetics, system durability, and ultimately the economic feasibility of solar hydrogen production.

### 3.3. Main water contaminants responsible for catalyst poisoning

The performance of photocatalysts for hydrogen production is strongly influenced by the quality of the feed water because various dissolved and suspended contaminants can interact with the catalyst surface, alter its electronic structure, or consume reactive species (Table 2). Kudo and Miseki^27^ reported that efficient photocatalytic water splitting generally requires highly purified water, as dissolved impurities can significantly reduce catalyst activity and long-term stability by blocking active sites and promoting charge-carrier recombination. Later, Hisatomi *et al.*^22^ emphasized that even trace amounts of foreign ions adsorbed on semiconductor surfaces can deteriorate charge separation efficiency and suppress hydrogen evolution, making water purity one of the key factors governing photocatalytic performance. Similarly, Wang and Domen^37^ highlighted that surface contamination and ion adsorption are major challenges preventing the practical application of particulate photocatalysts in real water systems.

**Table 2:** Main water contaminants responsible for catalyst poisoning in photocatalytic hydrogen production

Contaminant category	Representative contaminants	Reported effect on photocatalyst	Key reference
Heavy metal ions	Pb^2+^, Ni^2+^, Cu^2+^, Hg^2+^, Cr^6+^, Cd^2+^	Adsorb on active sites, act as electron traps/recombination centers, suppress H_2_ evolution (some ions such as Cd^2+^ may promote activity depending on catalyst system)	23
Natural organic matter (NOM)	Humic acid (HA), fulvic acid (FA)	Surface fouling, light shielding, scavenging of reactive species, reduced hydrogen evolution	24
Inorganic anions	Cl^−^, SO_4_^2−^, NO_3_^−^, PO_4_^3−^, HCO_3_^−^, CO_3_^2-^	Compete for adsorption sites; scavenge holes and hydroxyl radicals; reduce photocatalytic efficiency	23 and 24
Hardness ions	Ca^2+^, Mg^2+^	Cause mineral scaling and catalyst surface fouling during long-term operation	3
Suspended solids	Clay, silt, colloids, particulate matter	Reduce light penetration and physically cover catalyst surfaces	38

Among the various contaminants, heavy metal ions such as Pb^2+^, Ni^2+^, Cu^2+^, Hg^2+^, Cr^6+^, and Cd^2+^ are considered the most influential catalyst poisons because they strongly adsorb onto catalyst surfaces and modify the electronic properties of active sites (Fig. 2). Ahmed *et al.*^23^ reported that heavy metals can act as electron traps or recombination centers, leading to a significant decline in photocatalytic efficiency. In contrast, recent studies have shown that the effect depends on the specific metal ion and catalyst system.

**Fig. 2:**
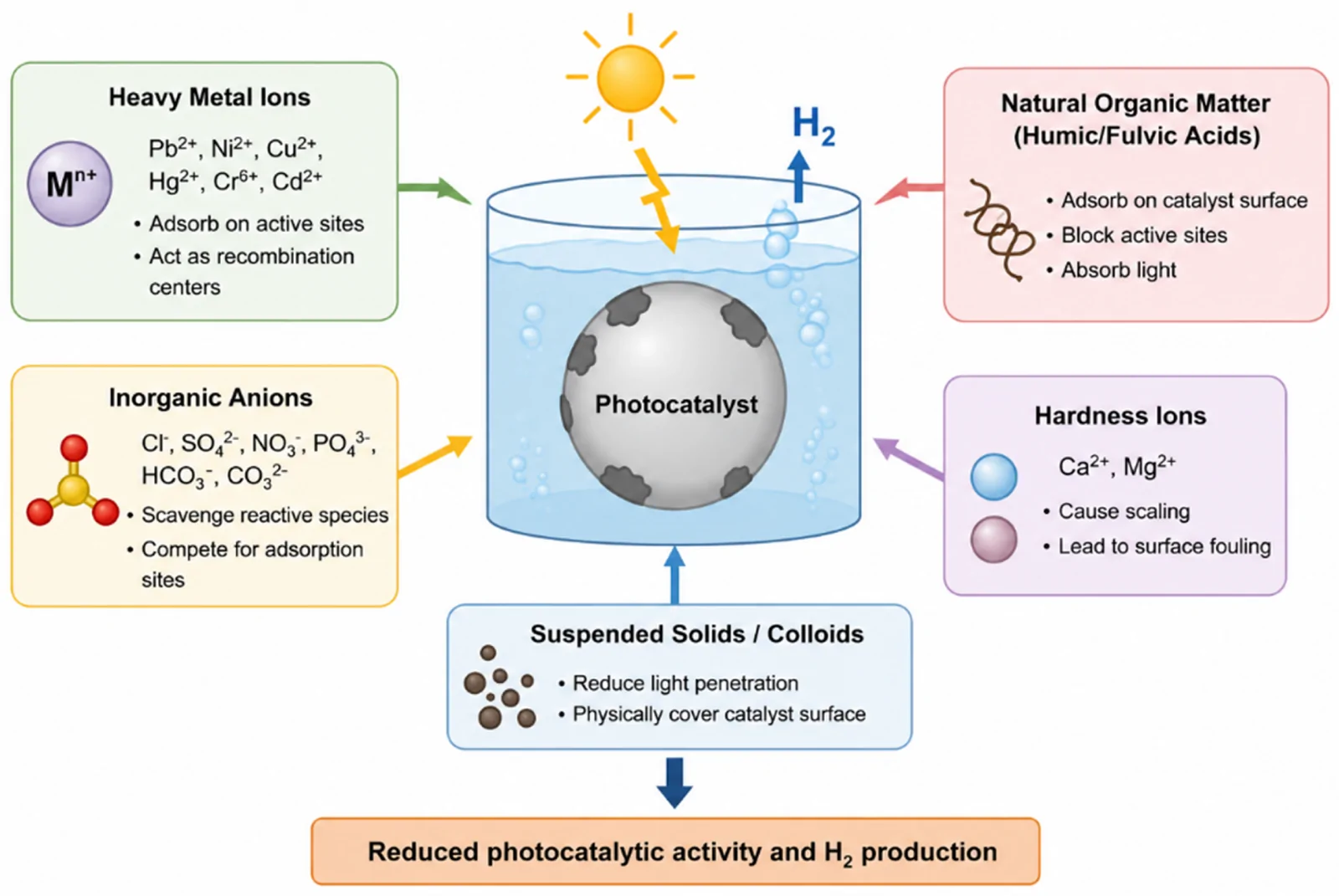
Main water contaminants responsible for catalyst poisoning.

Natural organic matter (NOM), particularly humic acid (HA) and fulvic acid (FA), is another important class of contaminants commonly found in surface waters. Chong *et al.*^24^ reported that humic substances adsorb strongly onto semiconductor surfaces, shield catalysts from incident light, and compete with water molecules for reactive oxygen species, thereby reducing photocatalytic efficiency. Likewise, Mills and Le Hunte^38^ demonstrated that dissolved organic compounds scavenge photogenerated holes and hydroxyl radicals, lowering overall photocatalytic reaction rates. In photocatalytic hydrogen production, humic acid has also been shown to decrease H_2_ evolution because of surface fouling and light attenuation.

Several inorganic anions, including chloride (Cl^−^), sulfate (SO_4_^2−^), nitrate (NO_3_^−^), phosphate (PO_4_^3−^), bicarbonate (HCO_3_^−^), and carbonate (CO_3_^2−^), also influence catalyst performance. Chong *et al.*^24^ reported that bicarbonate and carbonate ions readily scavenge hydroxyl radicals, while phosphate strongly adsorbs onto semiconductor surfaces and blocks catalytically active sites. It was further noted that chloride and sulfate ions compete with reactants for surface adsorption sites and alter surface charge characteristics, thereby decreasing photocatalytic efficiency.^23^ In addition to dissolved species, hardness ions (Ca^2+^ and Mg^2+^) and suspended solids contribute to catalyst deactivation during long-term operation. Wang and Domen^3^ explained that mineral precipitation caused by calcium- and magnesium-rich water can foul catalyst surfaces and reduce the accessibility of active sites. Furthermore, it was reported that suspended particles decrease light penetration and physically cover photocatalyst surfaces, thereby reducing photon utilization efficiency.^38^ Overall, the literature indicates that catalyst poisoning in photocatalytic hydrogen production is not caused by a single contaminant but rather by the combined effects of heavy metals, natural organic matter, inorganic ions, hardness ions, and suspended solids. Therefore, the development of contaminant-tolerant photocatalysts and the selection of suitable feedwater pretreatment strategies are essential for the large-scale implementation of photocatalytic water splitting. Table 3 shows quantitative comparison of photocatalytic H_2_ production under defined contaminant concentrations and realistic saline water.

**Table 3:** Quantitative comparison of photocatalytic H_2_ production under defined contaminant concentrations and realistic saline/water matrices

Photocatalyst	Water matrix/contaminant	Defined concentration/matrix	H_2_ evolution metric	AQY/AQE	Primary literature/DOI
Pt/g-C_3_N_4_/SrTiO_3_	Simulated dyeing wastewater; acid red 1	Acid red 1 = 50 mg L^−1^	471 µmol g^−1^ h^−1^	NR	39
Bi_3_TaO_7_/ZnIn_2_S_4_	Tetracycline-containing water	Tetracycline = 10 mg L^−1^; catalyst = 50 mg	13.7 µmol g^−1^ h^−1^	NR	40
25% Ni_5_P_4_/g-C_3_N_4_	Carbamazepine wastewater	Carbamazepine = 10 mg L^−1^; catalyst = 50 mg	40.1 mmol g^−1^ h^−1^	NR	41
20% NiCoP/g-C_3_N_4_	Saline water and natural seawater	3 wt% NaCl; natural seawater also tested	1827.4 µmol g^−1^ h^−1^ in 3 wt% NaCl; 1290.0 µmol g^−1^ h^−1^ in natural seawater	3.87% at 420 nm	42 and 43
CoSA-hCN	Artificial seawater and natural seawater	Artificial seawater: ASTM D1141-98; catalyst = 0.4 g L^−1^; TEOA = 10 vol%	6.4 ± 0.2 mmol g^−1^ h^−1^ in artificial seawater; 5.9 mmol g^−1^ h^−1^ in natural seawater	8.96% at 420 nm	44 and 45
UPy2 donor–π–acceptor carbon nitride	Natural seawater and simulated saline water	3.5 wt% NaCl or natural seawater; TEOA = 5–10 vol%; Pt = 3–5 wt%	44.50 mmol g^−1^ h^−1^ in seawater at 10 vol% TEOA; optimized 82.00 mmol g^−1^ h^−1^ at 5 vol% TEOA	4.77% at 450 nm in seawater; 1.46% in 3.5 wt% NaCl; 0.24% in DI water	43 and 45

### 3.4. What are sources of these contaminants in water

The contaminants responsible for catalyst poisoning originate from both natural processes and anthropogenic activities, and their concentrations vary depending on the water source, including groundwater, surface water, seawater, and municipal or industrial wastewater (Table 4). Qudsieh *et al.*^46^ reported that real water matrices contain a complex mixture of dissolved inorganic ions, NOM, suspended particles, and dissolved oxygen, all of which significantly influence photocatalytic performance by interacting with catalyst surfaces and reactive species. Their review emphasized that these constituents are ubiquitous in natural waters and must be considered when evaluating photocatalytic systems under practical conditions.

**Table 4:** Major sources of water contaminants affecting photocatalytic hydrogen production

Source	Major contaminants	Typical origin	Representative reference
Industrial discharges	Pb, Cd, Hg, Cr, Ni, Cu; organic chemicals	Mining, electroplating, textile, battery manufacturing, metal processing	33
Agricultural runoff	NO_3_^−^, PO_4_^3−^, pesticides, dissolved organic matter	Fertilizers, pesticides, livestock activities	29
Municipal sewage & domestic wastewater	Natural organic matter (NOM), nutrients, pharmaceuticals, suspended solids	Urban wastewater treatment plants and domestic discharge	29
Natural processes	Ca^2+^, Mg^2+^, HCO_3_^−^, CO_3_^2−^, humic substances	Rock weathering, soil erosion, decomposition of vegetation	29
Mining & metal processing	Heavy metals, acidity, sulfate	Mine drainage, ore extraction and smelting	47
Urban runoff	Suspended solids, chloride, hydrocarbons, metals	Stormwater, road dust, construction activities	29

Heavy metals such as Pb, Cd, Hg, Cr, Ni, Cu, as primarily originate from mining activities, metal processing, electroplating, battery manufacturing, textile industries, leather tanning, coal combustion, agricultural runoff, and improper disposal of industrial wastes (Fig. 3). Xu *et al.*^33^ reported that rapid industrialization and mining activities are the dominant sources of heavy-metal contamination in aquatic environments, while agricultural chemicals and municipal waste further increase metal concentrations in surface and groundwater. Likewise, Piwowarska *et al.*^47^ concluded that industrial effluents remain the largest contributor of toxic metals to freshwater ecosystems worldwide.

**Fig. 3:**
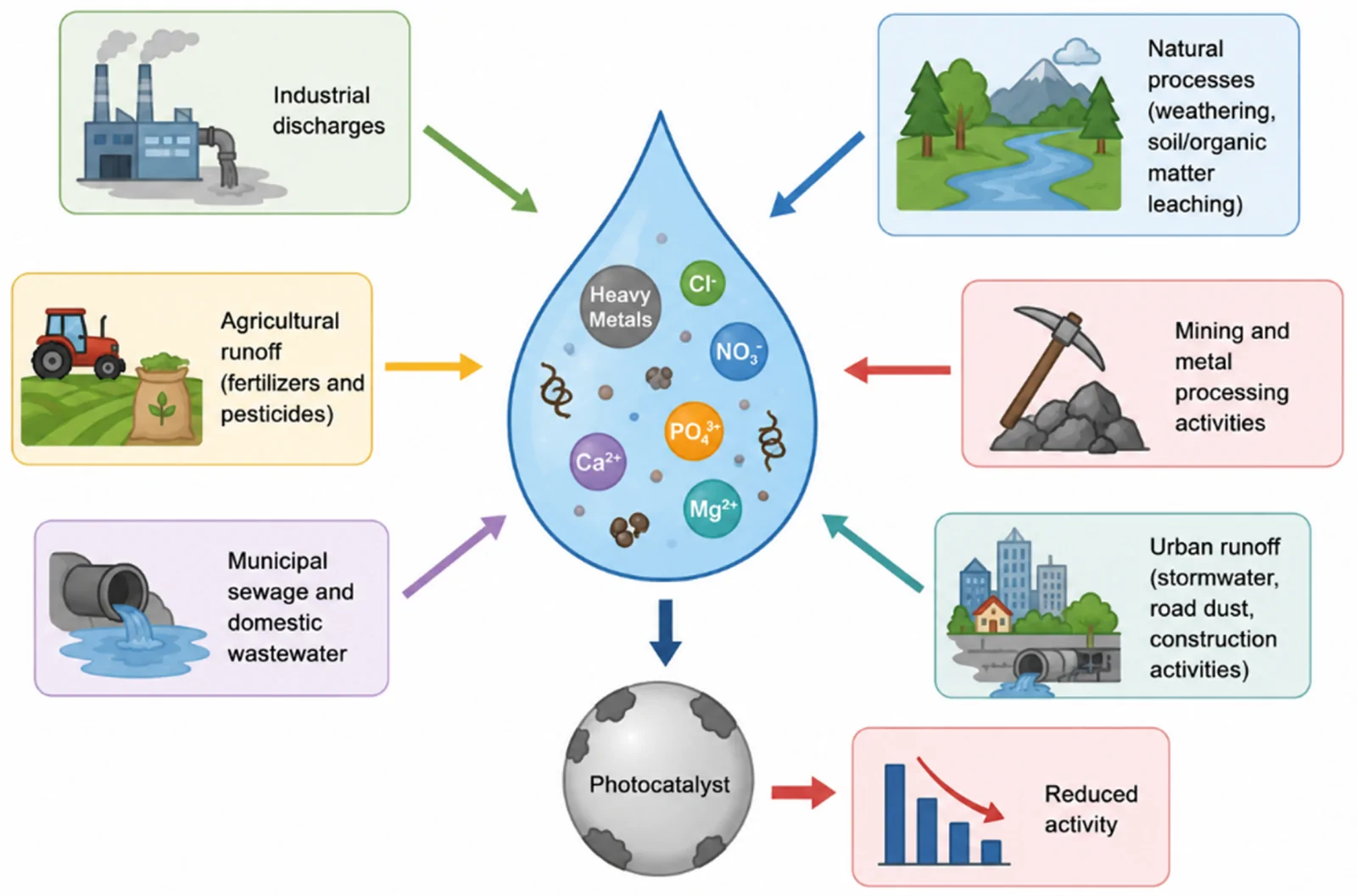
Various sources of water contaminants responsible for catalyst deactivation.

NOM, mainly humic acids and fulvic acids, originates from the decomposition of leaves, wood, algae, aquatic plants, and soil organic matter. It is reported that surface waters generally contain higher NOM concentrations because of continuous biological decomposition and runoff from surrounding vegetation. These organic compounds readily adsorb onto photocatalyst surfaces, reducing light absorption and blocking catalytic active sites.^29^ Inorganic anions, including chloride (Cl^−^), sulfate (SO_4_^2−^), nitrate (NO_3_^−^), phosphate (PO_4_^3−^), bicarbonate (HCO_3_^−^), and carbonate (CO_3_^2−^), originate from natural mineral weathering, seawater intrusion, fertilizer application, domestic sewage, and industrial discharges, these ions are among the most abundant constituents of natural waters and strongly influence photocatalytic reactions by scavenging reactive oxygen species or competing for catalyst adsorption sites.^29^ Hardness ions, particularly Ca^2+^ and Mg^2+^, are mainly derived from the dissolution of carbonate and silicate minerals such as limestone, dolomite, and gypsum, although industrial cooling water and groundwater abstraction can further increase hardness levels. Qudsieh *et al.*^29^ reported that these ions contribute to mineral scaling and catalyst surface fouling during prolonged photocatalytic operation, especially when untreated groundwater is used. Suspended solids and colloidal particles originate from soil erosion, sediment transport, urban stormwater runoff, wastewater discharge, and construction activities. It was observed that suspended particles increase turbidity, reduce light penetration, and physically cover catalyst surfaces, thereby lowering photon utilization efficiency and hydrogen production.^29^ Although many contaminants initially interact with photocatalysts through reversible adsorption or surface fouling, prolonged exposure or high contaminant concentrations can induce irreversible physicochemical changes. Reversible processes generally suppress photocatalytic activity temporarily and may be mitigated by catalyst washing, regeneration, or improved operating conditions. In contrast, irreversible deactivation involves permanent modifications to the catalyst structure or composition, including photocorrosion, crystal lattice degradation, cocatalyst dissolution, and surface reconstruction, leading to sustained losses in photocatalytic performance. Distinguishing these two categories is essential for developing effective mitigation strategies and designing water-quality-tolerant photocatalysts.

Photocatalytic hydrogen production under realistic water matrices remains significantly less explored than studies performed in ultrapure water (Table 5). Recent investigations have demonstrated that catalyst performance is strongly influenced by matrix composition, including salinity, hardness ions, natural organic matter, suspended solids, and trace metals. Although several advanced photocatalysts have exhibited excellent stability in seawater and wastewater, their long-term durability is generally verified only through post-reaction characterization techniques such as XPS, XRD, ICP-OES, TEM, SEM, XANES, and FTIR. These analyses reveal whether catalyst deactivation originates from contaminant adsorption, photocorrosion, cocatalyst dissolution, crystal structure degradation, or surface reconstruction. Consequently, integrating realistic water testing with comprehensive post-reaction characterization is essential for accurately evaluating catalyst durability and developing water-quality-tolerant photocatalysts suitable for practical hydrogen production.

**Table 5:** Representative photocatalytic hydrogen production studies using realistic water matrices and post-reaction characterization

Photocatalyst	Water matrix	Matrix composition	Operating conditions	Reaction duration	Post-reaction characterization	Reference
CoSA-hCN	Artificial seawater	ASTM D1141 artificial seawater	Xe lamp (>420 nm), TEOA	3 h + cyclic tests	XRD, FTIR, XANES	48
UPy_2_ donor–π–acceptor carbon nitride	Natural seawater	Natural seawater	AM 1.5G solar simulator	24 h	XRD, XPS	43
Atomically dispersed Ni photocatalyst	Real seawater	Natural seawater	Sacrificial-reagent-free photocatalysis	720 h (>15 cycles)	XPS, HAADF-STEM, ICP-OES	45
TiO_2_-based photocatalyst	Groundwater	Ca^2+^, Mg^2+^, HCO_3_^−^	Visible-light irradiation	6 h	XRD, SEM	42 and 49
g-C_3_N_4_ heterojunction	Municipal wastewater	Organic matter + inorganic ions	Visible-light irradiation	4 h	XPS, ICP-OES	50

## 4. Poisoning mechanisms

Water contaminants reduce the efficiency of photocatalytic hydrogen production through several physicochemical mechanisms rather than through a single poisoning pathway. These mechanisms interfere with photon absorption, charge separation, surface redox reactions, cocatalyst activity, and structural stability of photocatalysts. Although different contaminants exhibit different chemical properties, their detrimental effects generally occur through eight fundamental mechanisms: active-site blocking, competitive adsorption, charge carrier recombination, surface fouling, photocorrosion, crystal structure degradation, cocatalyst poisoning, and radical scavenging. Understanding these mechanisms is essential for designing durable photocatalysts capable of operating under realistic water conditions.

### 4.1. Active site blocking

Active-site blocking is one of the most direct poisoning mechanisms in photocatalytic water splitting (Fig. 4). Hydrogen evolution occurs on specific catalytic sites located on semiconductor surfaces or cocatalysts such as Pt, Rh, Ni, or MoS_2_. When dissolved contaminants strongly adsorb onto these active sites, they physically occupy the locations where proton reduction or water oxidation normally occurs. Consequently, the number of accessible catalytic sites decreases, leading to lower hydrogen production. Heavy metal ions including Pb^2+^, Ni^2+^, Hg^2+^, Cu^2+^, and Cd^2+^ possess high adsorption affinity toward metal oxides and noble-metal cocatalysts. These ions form stable surface complexes that inhibit electron transfer to adsorbed protons. Similarly, phosphate and carbonate ions may bind strongly through ligand exchange, permanently occupying hydroxylated surface sites. Kudo and Miseki^27^ reported that active-site poisoning significantly decreases the surface reaction rate even when the semiconductor continues to absorb sunlight efficiently. Therefore, catalyst activity becomes controlled by surface availability rather than photon absorption.

**Fig. 4:**
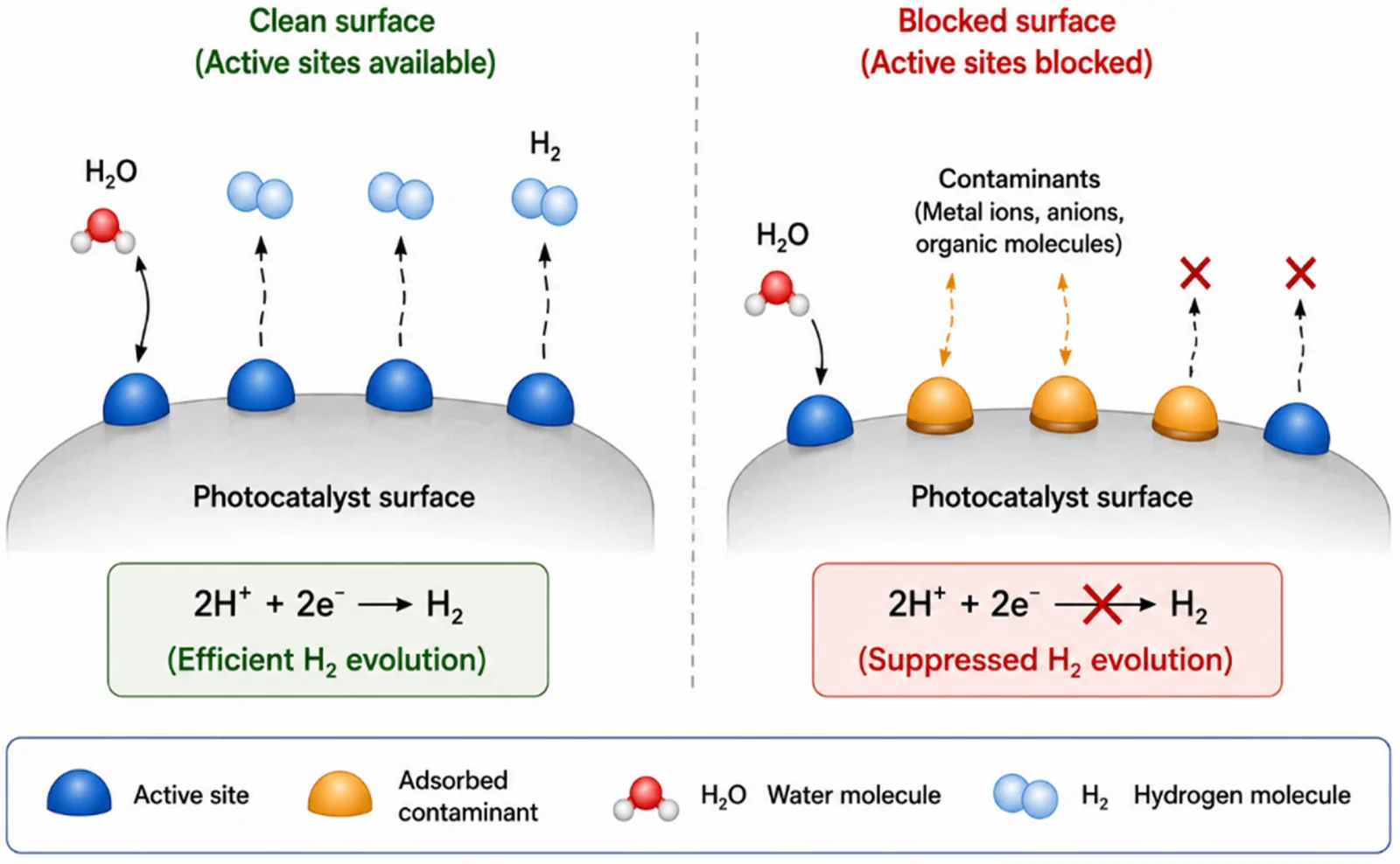
Schematic illustration of active-site blocking during photocatalytic hydrogen production.^27^

### 4.2. Competitive adsorption

Competitive adsorption occurs when dissolved contaminants compete with water molecules or reactants for adsorption onto photocatalyst surfaces. Because photocatalytic reactions begin with adsorption, any contaminant that preferentially adsorbs will reduce the availability of reaction sites for water splitting. Natural organic matter, chloride, sulfate, nitrate, bicarbonate, phosphate, and various organic pollutants exhibit considerable affinity toward semiconductor surfaces. Instead of allowing water molecules to adsorb and undergo oxidation, these contaminants occupy adsorption sites, thereby decreasing hydrogen evolution. Chong *et al.*^24^ demonstrated that competitive adsorption modifies surface chemistry and suppresses the formation of reactive intermediates required for efficient photocatalysis. Unlike active-site blocking, competitive adsorption is often partially reversible and depends strongly on contaminant concentration, pH, and ionic strength.

### 4.3. Charge carrier recombination

Efficient photocatalytic hydrogen production requires rapid separation of photogenerated electrons and holes before recombination occurs. Unfortunately, many contaminants introduce additional recombination centers that dramatically shorten carrier lifetime. Transition metal ions such as Fe^3+^, Cu^2+^, Cr^3+^, and Mn^2+^ may repeatedly capture and release electrons, acting as electron–hole recombination centers. Surface defects generated by contaminant adsorption also provide localized electronic states where electrons and holes recombine without participating in hydrogen evolution. Hisatomi *et al.*^11^ emphasized that recombination remains one of the largest factors limiting solar-to-hydrogen efficiency because only a small fraction of photogenerated charge carriers ultimately participate in surface reactions.

### 4.4. Surface fouling

Surface fouling involves the accumulation of suspended solids, natural organic matter, microorganisms, biofilms, colloidal particles, or mineral scales on photocatalyst surfaces. Unlike molecular adsorption, fouling forms a continuous physical layer that covers the catalyst.

This deposited layer reduces light penetration, blocks active sites, increases diffusion resistance, and limits contact between water molecules and catalytic surfaces. Humic substances frequently produce organic coatings on TiO_2_ and other oxide photocatalysts, whereas calcium and magnesium ions may generate scaling deposits under alkaline conditions. According to Chong *et al.*^24^ surface fouling becomes increasingly important during long-term operation using river water, groundwater, and wastewater.

### 4.5. Photocorrosion

Photocorrosion is a degradation process in which the photocatalyst itself undergoes oxidation or reduction instead of catalyzing water splitting. Photogenerated holes oxidize lattice atoms, while electrons reduce metal ions within the semiconductor structure. Materials such as CdS, ZnO, Cu_2_O, and several metal sulfides are particularly susceptible because their lattice atoms are thermodynamically unstable under illumination. Continuous photocorrosion gradually dissolves the catalyst, decreases crystallinity, and permanently reduces hydrogen production. Maeda and Domen^34^ identified photocorrosion as one of the principal barriers preventing the commercialization of visible-light photocatalysts.

### 4.6. Crystal structure degradation

Certain contaminants penetrate into the semiconductor lattice or react with lattice atoms, producing phase transformation, amorphization, lattice distortion, oxygen vacancy redistribution, or crystal defects. These structural changes modify electronic band structures, reduce charge mobility, alter surface energy, and decrease catalytic activity. Long-term exposure to contaminated water may also induce particle fragmentation or crystal growth.

Kranz and Wächtler^35^ demonstrated that XRD, XPS, and TEM analyses frequently reveal progressive structural degradation after prolonged photocatalytic operation.

### 4.7. Co-catalyst poisoning

Most efficient photocatalysts contain cocatalysts such as Pt, Rh, Ru, Ni, Co, or MoS_2_ that provide active sites for hydrogen evolution. Because hydrogen evolution primarily occurs on these cocatalysts, poisoning them has a much greater impact than poisoning the semiconductor itself. Sulfur compounds, chloride ions, heavy metals, and organic sulfur species strongly adsorb onto cocatalyst surfaces, preventing proton adsorption and electron transfer. Sulfur poisoning of platinum is a classical example in heterogeneous catalysis and similarly affects photocatalytic hydrogen production. Osterloh^51^ reported that cocatalyst poisoning substantially decreases hydrogen evolution despite maintaining semiconductor crystallinity.

### 4.8. Radical scavenging

Reactive oxygen species including hydroxyl radicals (˙OH), superoxide radicals (O_2_˙^−^), and photogenerated holes play essential roles during photocatalytic reactions. Many dissolved contaminants consume these reactive species before they participate in water oxidation. Carbonate, bicarbonate, chloride, nitrate, humic substances, and dissolved organic compounds rapidly react with hydroxyl radicals, decreasing oxidative capacity and modifying reaction pathways. Organic contaminants may also directly consume photogenerated holes. Chong *et al.*^24^ demonstrated that radical scavenging becomes increasingly important in natural waters because multiple dissolved ions simultaneously compete for reactive intermediates, thereby reducing photocatalytic efficiency.

## 5. Water quality-cost relationship

Water quality is increasingly recognized as an important economic factor influencing the commercial viability of photocatalytic hydrogen production. While most techno-economic analyses focus on catalyst cost, reactor design, and solar-to-hydrogen (STH) efficiency, the quality of the feedwater can substantially affect both capital and operating expenses (Fig. 5). Maurya *et al.*^36^ developed a techno-economic assessment for photocatalytic water splitting and reported that capital investment and labor account for nearly 75% of the levelized cost of hydrogen (LCOH), whereas material-related costs contribute 13–29%. Although their analysis assumed controlled water conditions, the authors emphasized that catalyst lifetime and operational stability strongly influence hydrogen production costs, implying that catalyst poisoning caused by poor-quality water would further increase the LCOH (Table 6).^36^

**Fig. 5:**
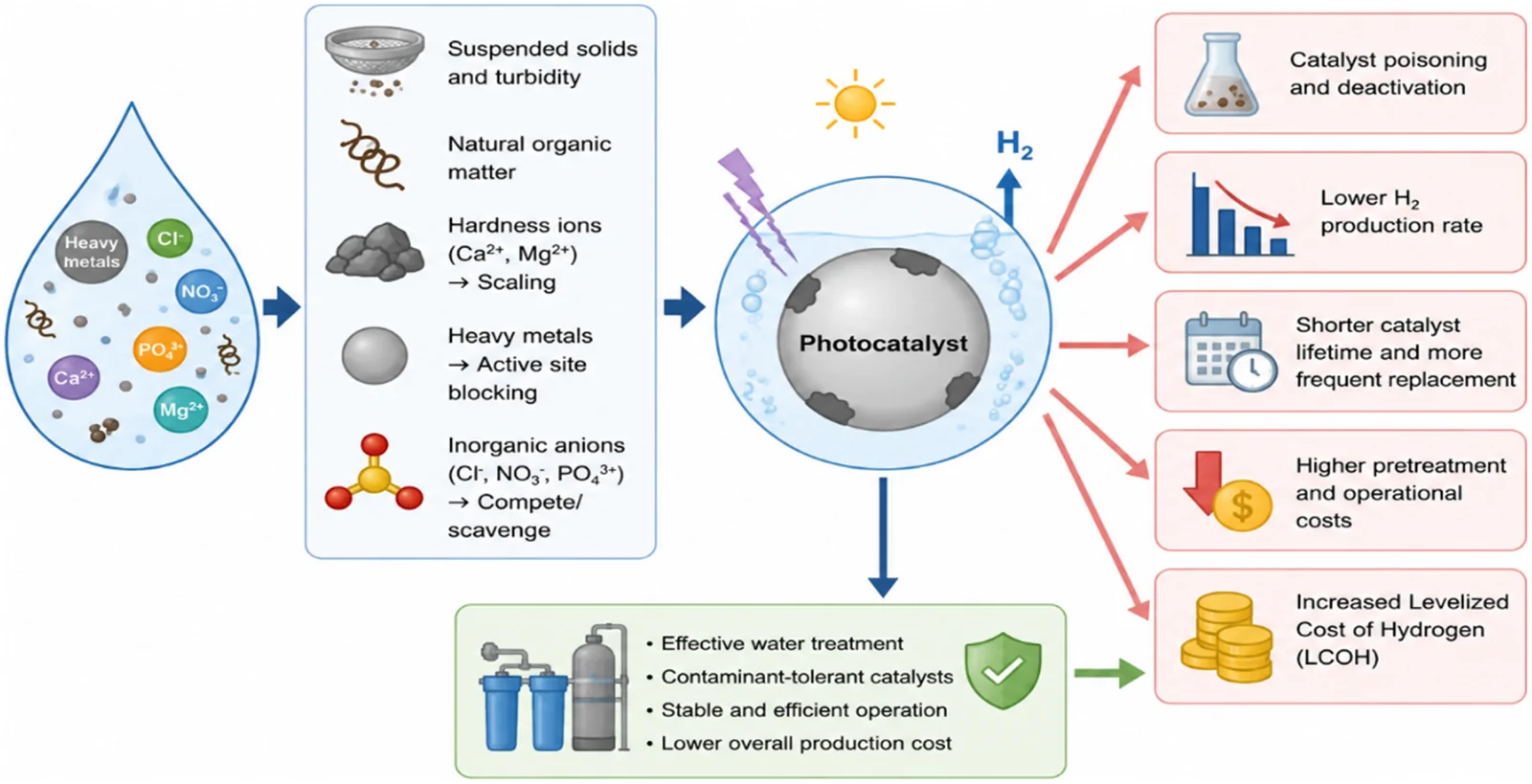
Economic relationship of water quality during hydrogen energy production.

**Table 6:** Quantitative economic implications of water quality and catalyst poisoning n hydrogen production

Parameter	Typical value	Economic implication	Representative reference
Theoretical water consumption	9 L per kg H_2_	Minimum stoichiometric water requirement	36
Practical water consumption	12–25 L per kg H_2_	Additional water for purification, cooling and auxiliary operations	36
Typical water purity requirement	>1 MΩ cm (often >5 MΩ cm for PEM)	Higher water quality increases pretreatment cost	36
Pretreatment processes	Filtration, RO, ion exchange, deionization	Increase CAPEX and OPEX	25
Photocatalytic H_2_ cost (LCOH)	US$ 4.9–7.8 per kg H_2_	Depends strongly on catalyst lifetime and efficiency	36
Capital cost contribution	≈75%	Infrastructure and labor dominate investment	36
Material-related cost	13–29%	Catalyst replacement affects production cost	36
Catalyst replacement	Higher in contaminated water	Higher maintenance and catalyst costs	36
Natural *vs.* ultrapure water	Lower efficiency in real water	Pretreatment often required	29
Overall impact	Lower lifetime, lower H_2_ yield, higher LCOH	Economic barrier to commercialization	29

The use of contaminated water introduces additional economic burdens because pretreatment processes such as filtration, sedimentation, activated carbon adsorption, ion exchange, softening, reverse osmosis, or deionization are often required before photocatalytic hydrogen production. These processes consume energy, chemicals, and maintenance resources, thereby increasing both capital expenditure (CAPEX) and operating expenditure (OPEX). Gunawan *et al.*^25^ highlighted that improvements in photocatalytic efficiency alone are insufficient for achieving economically competitive hydrogen production because upstream water treatment and catalyst replacement costs must also be considered when evaluating industrial-scale systems. They further demonstrated that hydrogen production costs decrease significantly only when catalyst performance and system efficiency are maintained over long operating periods.

Poor water quality also accelerates catalyst deactivation through heavy-metal adsorption, natural organic matter deposition, mineral scaling, and inorganic ion accumulation. These processes shorten catalyst lifetime, increase regeneration frequency, and require more frequent catalyst replacement, directly increasing production costs. Maurya *et al.* concluded that photocatalyst durability is one of the key economic parameters affecting LCOH because unstable catalysts require repeated synthesis, regeneration, or replacement during plant operation. Similarly, it was demonstrated that even moderate reductions in catalyst stability substantially affect overall hydrogen production economics due to increased maintenance requirements and reduced reactor productivity.^36^

Another important economic consequence is the reduction in hydrogen productivity caused by catalyst poisoning. Lower hydrogen evolution rates decrease reactor throughput while fixed costs including reactor infrastructure, land, and labor remain nearly unchanged, thereby increasing the cost per kilogram of hydrogen. It was reviewed multiple techno-economic studies and showed that improvements in STH efficiency markedly reduce hydrogen production costs, whereas catalyst deactivation has the opposite effect by lowering reactor productivity. Likewise, Maurya *et al.* reported LCOH values ranging from approximately US$ 4.9–7.8 per kg H_2_ for different photocatalytic systems under controlled operating conditions, indicating that any reduction in catalyst performance caused by poor water quality would further increase these costs.^25^

Table 7 highlights the economic trade-offs between feedwater pretreatment and catalyst durability in photocatalytic hydrogen production. Although advanced pretreatment technologies such as reverse osmosis, ion exchange, and deionization increase capital and operating expenditures, they substantially reduce the concentration of dissolved contaminants responsible for catalyst poisoning. In contrast, inadequate pretreatment accelerates active-site blocking, surface fouling, mineral scaling, and photocorrosion, resulting in more frequent catalyst regeneration or replacement, particularly for photocatalysts containing expensive noble metals. Consequently, an optimal balance between water pretreatment and catalyst durability is essential for minimizing the levelized cost of hydrogen (LCOH). Recent techno-economic analyses suggest that integrating cost-effective pretreatment with contaminant-tolerant photocatalysts provides the most economically sustainable pathway for large-scale green hydrogen production.

**Table 7:** Comparison of water pretreatment costs and catalyst replacement costs in hydrogen production systems based on representative literature^a^

Economic factor	Typical CAPEX/OPEX or cost	Effect on hydrogen production	Economic implication	Representative references
Coarse filtration & sediment removal	Low CAPEX; OPEX < 2% of total water treatment cost	Removes suspended solids and turbidity	Reduces catalyst surface fouling and light scattering	29 and 30
Activated carbon adsorption	Moderate CAPEX; periodic adsorbent replacement	Removes NOM, humic substances and organics	Minimizes catalyst poisoning caused by organic fouling	24 and 30
Ion exchange	Moderate CAPEX; resin regeneration required	Removes hardness ions (Ca^2+^, Mg^2+^) and heavy metals	Reduces scaling and active-site poisoning	30 and 31
Reverse osmosis (RO)	High CAPEX; energy-intensive OPEX	Produces high-purity water by removing dissolved salts and metals	Improves catalyst stability but increases operating cost	21 and 30
Deionization (DI)	Moderate-to-high CAPEX; resin replacement	Produces ultrapure water for laboratory or PEM systems	Maximizes catalyst lifetime but increases pretreatment expenses	21 and 36
Catalyst regeneration	Typically 10–30% of fresh catalyst cost	Restores part of catalyst activity after poisoning	Lower cost than complete replacement; repeated regeneration may reduce activity	36
Catalyst replacement	Depends on catalyst composition (especially Pt or Rh)	Required after irreversible poisoning or photocorrosion	Significantly increases material cost and LCOH	36
Integrated pretreatment + contaminant-tolerant catalyst	Higher initial investment but lower long-term operating cost	Reduces contaminant loading and extends catalyst lifetime	Lowest overall lifetime cost and improved economic sustainability	25 and 36

a Abbreviations: CAPEX = capital expenditure; OPEX = operating expenditure; LCOH = levelized cost of hydrogen; RO = reverse osmosis; DI = deionized water; NOM = natural organic matter.

From a broader sustainability perspective, the economic impact of water quality extends beyond the photocatalytic reactor itself. Water treatment infrastructure, freshwater availability, wastewater management, and environmental compliance all contribute to the overall cost of green hydrogen production. Recent reviews emphasize that integrating contaminant-tolerant photocatalysts with low-cost water treatment technologies represents one of the most promising strategies for reducing hydrogen production costs while enabling the direct utilization of natural waters and wastewater. Consequently, improving catalyst resistance to water contaminants can provide simultaneous economic and environmental benefits by lowering pretreatment requirements, extending catalyst lifetime, and reducing the levelized cost of hydrogen.

The levelized cost of hydrogen (LCOH) is highly sensitive to feedwater quality because pretreatment requirements directly affect both capital investment and operating expenditures. In an ideal scenario using ultrapure water, pretreatment costs are minimal and catalyst lifetime is maximized, resulting in the lowest LCOH. However, practical hydrogen production is expected to rely increasingly on freshwater, groundwater, seawater, and reclaimed wastewater, all of which require progressively more intensive pretreatment. Techno-economic studies indicate that additional unit operations such as filtration, ion exchange, reverse osmosis, deionization, and advanced oxidation can increase water treatment costs while simultaneously improving catalyst durability. A simplified sensitivity analysis suggests that low-complexity pretreatment (*e.g.*, filtration and softening) may increase the LCOH by approximately 5–10%, whereas moderate pretreatment involving ion exchange or partial desalination can increase the LCOH by 10–20%. In contrast, high-intensity pretreatment for seawater or wastewater, incorporating reverse osmosis, deionization, and advanced oxidation processes, may increase the LCOH by 20–30%, depending on local energy prices, membrane replacement frequency, and chemical consumption. Nevertheless, these additional pretreatment costs may be partially or fully offset by extending catalyst lifetime, reducing catalyst replacement frequency, and maintaining stable hydrogen production under realistic operating conditions. Consequently, future techno-economic optimization should simultaneously evaluate pretreatment intensity, catalyst durability, and contaminant tolerance rather than considering these parameters independently.

## 6. Testing of water for water quality

Understanding the influence of water contaminants on photocatalytic hydrogen production requires a combination of physicochemical, spectroscopic, microscopic, and water quality analyses to identify contaminant species, monitor catalyst degradation, and elucidate poisoning mechanisms (Fig. 6). Kranz and Wächtler^35^ reported that structural characterization techniques such as X-ray diffraction (XRD) are widely used to detect phase transformations, crystallinity changes, and structural degradation of photocatalysts after exposure to contaminated water, while scanning electron microscopy (SEM) and transmission electron microscopy (TEM/HR-TEM) reveal morphological changes, particle agglomeration, and surface fouling caused by inorganic and organic deposits. Furthermore, energy-dispersive X-ray spectroscopy (EDS) provides elemental mapping of deposited contaminants, whereas X-ray photoelectron spectroscopy (XPS) is one of the most powerful techniques for determining changes in oxidation states, chemical bonding, and the adsorption of poisoning species on catalyst surfaces. To quantify dissolved contaminants in water before and after photocatalytic reactions, inductively coupled plasma-optical emission spectroscopy (ICP-OES) and ICP-mass spectrometry (ICP-MS) is routinely employed for trace metal analysis, while ion chromatography (IC) measures inorganic anions such as chloride, nitrate, phosphate, and sulfate.

**Fig. 6:**
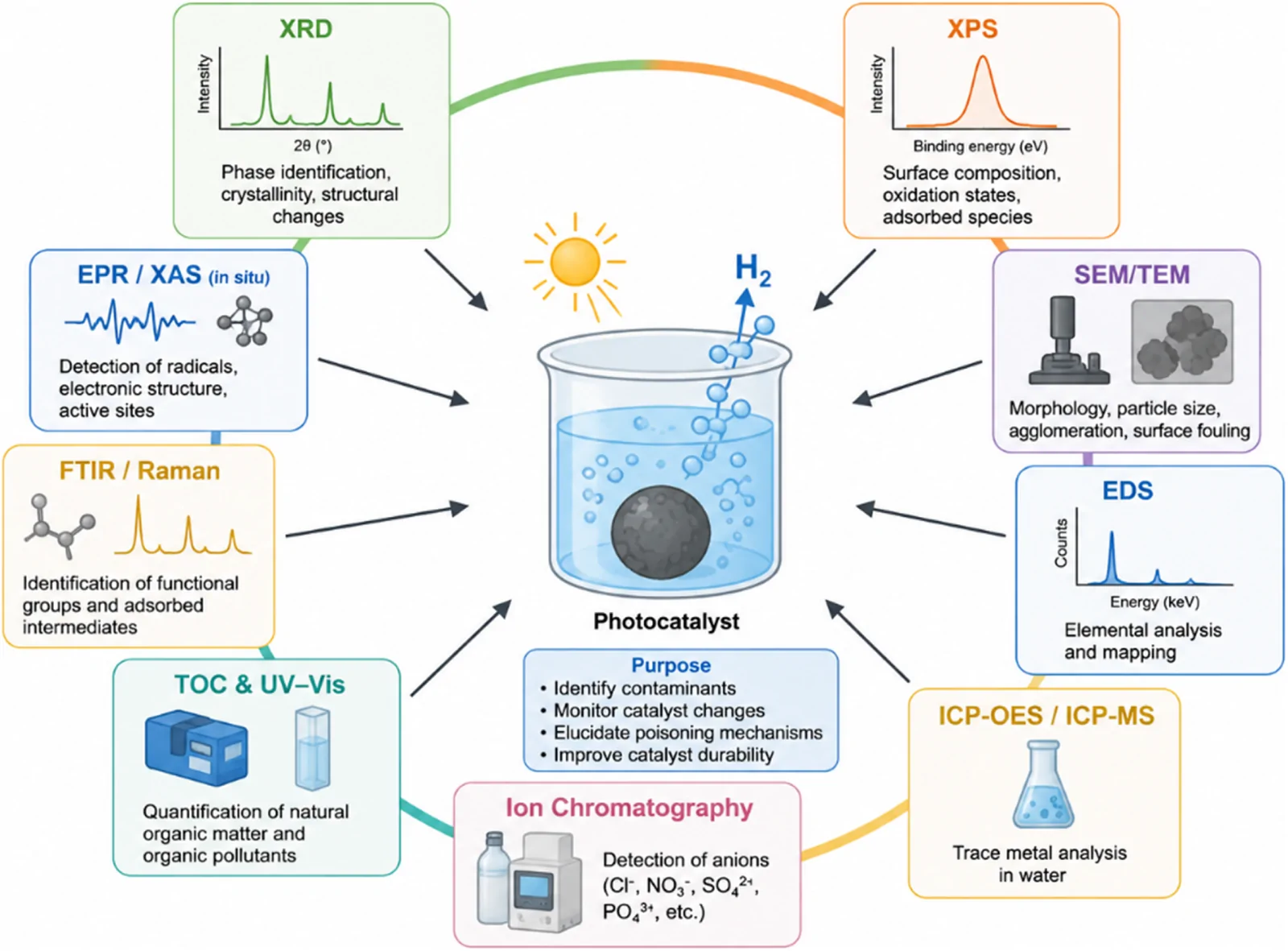
Various methods available for measurements of poisoning contaminants in water.

Total organic carbon (TOC) analysis and UV-Vis spectroscopy are commonly used to determine natural organic matter and dissolved organic pollutants that compete with photocatalytic reactions. For mechanistic investigations, Mu *et al.*^52^ emphasized that *in situ* Fourier-transform infrared spectroscopy (FTIR), Raman spectroscopy, electron paramagnetic resonance (EPR), and X-ray absorption spectroscopy (XAS) enable real-time monitoring of adsorbed intermediates, surface poisoning, defect formation, and charge-transfer processes during photocatalytic reactions. More recently, Khan and Ersoz^53^ highlighted that integrating *operando* spectroscopy, advanced surface analysis, and theoretical modeling provides molecular-level insight into contaminant adsorption, active-site blocking, and catalyst deactivation, thereby facilitating the rational design of contaminant-tolerant photocatalysts for sustainable hydrogen production.

### 6.1. Advanced characterization methods for understanding water-induced photocatalyst poisoning

A comprehensive understanding of water quality-induced catalyst poisoning requires characterization techniques capable of identifying not only structural changes but also dynamic surface processes, electronic interactions, and contaminant–catalyst interfaces during photocatalytic operation. Conventional characterization methods such as X-ray diffraction (XRD), scanning electron microscopy (SEM), transmission electron microscopy (TEM), X-ray photoelectron spectroscopy (XPS), and UV-Vis spectroscopy provide valuable information regarding crystal structure, morphology, elemental composition, and surface chemical states. However, these *ex situ* techniques often fail to capture the transient processes occurring during photocatalytic reactions. Therefore, advanced *operando*, spectroscopic, microscopic, electrochemical, and computational approaches are increasingly being employed to elucidate the mechanisms responsible for catalyst poisoning under realistic water-splitting conditions.

#### 6.1.1. *Operando* spectroscopy

*Operando* spectroscopy represents one of the most powerful approaches for studying photocatalysts under actual reaction conditions by simultaneously monitoring catalyst structure, surface intermediates, and catalytic performance during illumination. Unlike conventional *ex situ* measurements performed before and after reaction, *operando* techniques provide real-time information about dynamic changes occurring at catalyst surfaces. Techniques such as *operando* infrared spectroscopy (FTIR), Raman spectroscopy, X-ray absorption spectroscopy (XAS), and ambient-pressure XPS (AP-XPS) can identify adsorbed contaminants, surface intermediates, oxidation-state changes, and catalyst reconstruction processes. For water-contaminant-induced poisoning, *operando* spectroscopy is particularly useful for detecting the adsorption of heavy metals, organic molecules, and inorganic ions on active sites and determining whether these interactions are reversible or permanent. Kranz and Wächtler^35^ emphasized that *operando* characterization is essential for understanding photocatalytic mechanisms because many catalyst transformations occur only under illumination and reaction conditions. Similarly, Henderson *et al.*^54^ demonstrated that *in situ* spectroscopic methods provide direct evidence of surface adsorption and reaction intermediates that cannot be obtained from conventional characterization.

#### 6.1.2. Synchrotron X-ray absorption spectroscopy (XAS)

Synchrotron-based X-ray absorption spectroscopy, including X-ray absorption near-edge structure (XANES) and extended X-ray absorption fine structure (EXAFS), provides element-specific information about oxidation states, coordination environments, and local atomic structures. For example, transition-metal contaminants such as Fe, Ni, Cu, and Mn may interact strongly with semiconductor surfaces and alter charge-transfer pathways. XAS enables direct observation of these interactions at the atomic scale. Synchrotron XAS is particularly valuable for investigating catalytic materials because it can monitor structural evolution even when catalysts are poorly crystalline or contain highly dispersed active species.^55^

#### 6.1.3. Kelvin probe force microscopy (KPFM)

Kelvin probe force microscopy is an advanced scanning probe technique used to measure surface potential distribution and charge-transfer behavior at nanoscale resolution. Since photocatalytic performance strongly depends on charge separation and migration, KPFM provides direct information regarding electron accumulation, surface electric fields, and photogenerated charge movement. In poisoning studies, KPFM can identify how adsorbed contaminants modify surface potentials and influence electron transfer pathways. For example, contaminant adsorption may create surface trap states that accelerate electron–hole recombination or alter band bending. Zhang *et al.*^56^ demonstrated that KPFM is an effective tool for visualizing charge separation behavior in semiconductor photocatalysts and understanding the relationship between surface electronic properties and photocatalytic activity.

#### 6.1.4. Electrochemical impedance spectroscopy (EIS)

Electrochemical impedance spectroscopy (EIS) is widely applied to evaluate interfacial charge-transfer resistance, carrier mobility, and recombination behavior. In photocatalytic water splitting, a smaller charge-transfer resistance generally indicates faster electron transport and improved catalytic activity. Nyquist plots obtained from EIS measurements provide information about charge-transfer kinetics and catalyst/electrolyte interfaces. Low-frequency impedance increases are commonly associated with surface blockage and slower interfacial reactions caused by contaminant adsorption. According to Osterloh,^51^ electrochemical measurements are essential for separating the effects of light absorption, charge generation, and surface catalytic reactions.

#### 6.1.5. Photoluminescence (PL) spectroscopy

Photoluminescence spectroscopy is commonly used to investigate electron–hole recombination processes in semiconductor photocatalysts. Generally, strong PL emission indicates rapid recombination, whereas reduced PL intensity suggests improved charge separation. In poisoning studies, changes in PL intensity can reveal whether contaminants introduce additional recombination centers or modify defect states. For example, adsorption of metal ions may create trap states that increase non-radiative recombination, decreasing the availability of electrons for hydrogen evolution. Hisatomi *et al.*^22^ highlighted that understanding charge-carrier dynamics is essential for improving photocatalytic efficiency because recombination significantly limits solar-to-hydrogen conversion.

#### 6.1.6. Time-resolved fluorescence and transient spectroscopy

Time-resolved fluorescence techniques, including time-resolved photoluminescence (TRPL) and transient absorption spectroscopy, provide information about carrier lifetimes and migration pathways. During contaminant poisoning, reduced carrier lifetime indicates enhanced recombination caused by surface defects or adsorbed species. Conversely, longer carrier lifetimes suggest improved charge separation.

#### 6.1.7. Density functional theory (DFT) calculations

Density functional theory (DFT) has become an important computational approach for explaining contaminant–photocatalyst interactions at the atomic level. DFT calculations can predict: For example, DFT can explain why Pb^2+^ or phosphate ions preferentially bind to oxygen vacancies or metal sites, leading to active-site blocking or altered charge-transfer pathways. Kubacka *et al.*^57^ emphasized that combining DFT calculations with experimental characterization provides molecular-level understanding of photocatalytic mechanisms and guides rational catalyst design.

#### 6.1.8. Molecular dynamics (MD) simulations

Molecular dynamics simulations complement DFT studies by describing dynamic interactions between catalysts, water molecules, and contaminants over longer timescales. For realistic water environments containing multiple ions and organic molecules, MD simulations provide valuable information about complex interfacial phenomena that are difficult to observe experimentally. Kubacka *et al.*^57^ demonstrated that molecular simulations are useful for predicting adsorption behavior and transport properties of contaminants at catalyst–water interfaces.

## 7. Artificial intelligence and machine learning for water quality-aware photocatalytic hydrogen production

Artificial intelligence (AI) and machine learning (ML) are rapidly transforming materials science and catalysis by enabling data-driven discovery, optimization, and predictive analysis. Traditionally, the development of photocatalysts has relied on trial-and-error experimentation combined with density functional theory (DFT) calculations, which are often time-consuming and computationally expensive. Recent advances in AI have introduced powerful algorithms capable of learning complex relationships between catalyst composition, electronic structure, synthesis parameters, reaction conditions, and photocatalytic performance. As Wang *et al.*^58^ highlighted, machine learning models can significantly accelerate photocatalyst discovery by predicting catalytic activity, hydrogen evolution rates, band gaps, and optimal synthesis conditions from large experimental and computational datasets, thereby reducing experimental cost and shortening development time.

### 7.1. Predicting catalyst poisoning

One of the emerging applications of AI in photocatalysis is the prediction of catalyst poisoning caused by dissolved contaminants. Large experimental databases containing information on contaminant concentration, catalyst composition, reaction conditions, and hydrogen evolution performance can be used to train supervised learning algorithms such as random forests, support vector machines, gradient boosting, and artificial neural networks. These models can identify complex nonlinear relationships between contaminant properties and catalyst deactivation that are difficult to recognize using conventional statistical methods. Machine learning can also rank the relative importance of poisoning mechanisms, estimate catalyst lifetime, and predict performance degradation under different water chemistries. More recently, hybrid AI-DFT frameworks have been proposed to predict contaminant adsorption energies, active-site poisoning, and electronic structure modifications before experimental validation, thereby accelerating the development of poisoning-resistant photocatalysts.

### 7.2. Water quality prediction

Machine learning has also become an effective tool for predicting water quality, which directly influences photocatalytic hydrogen production. Modern AI models integrate sensor data, satellite observations, meteorological information, and historical water quality records to estimate concentrations of heavy metals, nutrients, natural organic matter, suspended solids, salinity, and pH. These predictive models enable continuous assessment of feedwater suitability before hydrogen production and provide early warnings when contaminant levels exceed acceptable limits. For large-scale hydrogen plants utilizing natural water resources or treated wastewater, AI-based water quality forecasting can support adaptive pretreatment strategies, reduce unnecessary purification costs, and improve long-term catalyst stability. Integrating water-quality prediction with photocatalytic reactor operation represents an important step toward intelligent hydrogen production systems.^59^

### 7.3. AI-guided catalyst design

Machine learning is increasingly used to accelerate the discovery and optimization of photocatalysts by predicting structure–property relationships from extensive experimental and theoretical databases. Instead of experimentally synthesizing thousands of materials, AI algorithms can rapidly screen candidate semiconductors based on descriptors such as band-gap energy, conduction- and valence-band positions, adsorption energy, surface area, crystallinity, defect density, and cocatalyst composition. Wang *et al.* demonstrated that intelligent algorithms can accurately predict photocatalyst performance while identifying key material descriptors governing photocatalytic activity. When combined with high-throughput DFT calculations, machine learning enables inverse catalyst design, in which target properties are specified first and candidate materials are subsequently generated computationally. Such approaches have the potential to identify contaminant-tolerant photocatalysts with enhanced resistance to surface poisoning and improved long-term stability.^60^

### 7.4. Process optimization

Beyond catalyst development, AI provides powerful tools for optimizing photocatalytic reaction conditions. Photocatalytic hydrogen production depends on numerous interacting variables, including catalyst loading, light intensity, irradiation wavelength, pH, sacrificial reagent concentration, cocatalyst loading, temperature, contaminant concentration, and reaction time. Conventional optimization methods typically vary one parameter at a time and often overlook complex interactions between variables. Machine learning algorithms can simultaneously analyze multidimensional datasets to determine optimal operating conditions that maximize hydrogen evolution while minimizing catalyst deactivation. Reinforcement learning, Bayesian optimization, and genetic algorithms are increasingly being applied to optimize reactor design, reaction conditions, and operational strategies. These approaches reduce experimental workload while improving reproducibility and overall process efficiency.^61^

### 7.5. Digital twins

Digital twin technology represents one of the newest developments in intelligent process engineering. A digital twin is a virtual representation of a physical photocatalytic reactor that continuously receives real-time operational data from sensors and predicts future system behavior using physics-based and AI-driven models. For photocatalytic hydrogen production, digital twins can integrate information on water quality, catalyst condition, light intensity, hydrogen production rate, and reactor operating parameters to simulate system performance in real time. Such models can identify catalyst deactivation before significant performance losses occur, recommend optimal operating conditions, schedule catalyst regeneration, and predict maintenance requirements. In future hydrogen production facilities, digital twins could provide continuous monitoring of catalyst poisoning, estimate remaining catalyst lifetime, and optimize pretreatment strategies according to changing water quality, thereby improving reliability and reducing operational costs. Although digital twin applications in photocatalytic water splitting remain at an early stage, their successful implementation in chemical process industries suggests considerable potential for intelligent and autonomous hydrogen production systems.^62^

## 8. Removal of water contaminants prior to photocatalytic hydrogen production

### 8.1. Importance of water pretreatment

Water pretreatment is an essential step for maintaining the efficiency and long-term stability of photocatalytic hydrogen production systems. Although ultrapure or deionized water is commonly employed in laboratory investigations to eliminate the influence of dissolved contaminants, large-scale hydrogen production is expected to utilize natural water, reclaimed wastewater, groundwater, seawater, or industrial effluents, all of which contain a wide variety of inorganic ions, heavy metals, natural organic matter (NOM), microorganisms, and suspended solids. These contaminants can adsorb onto catalyst surfaces, occupy active sites, induce photocorrosion, promote electron–hole recombination, or alter the surface chemistry of photocatalysts, ultimately decreasing hydrogen evolution efficiency. Consequently, effective water pretreatment is necessary not only to improve photocatalytic performance but also to extend catalyst lifetime and reduce operational costs associated with catalyst replacement. The required level of pretreatment depends on both the source water and the tolerance of the photocatalyst. For highly sensitive photocatalysts, advanced purification processes may be required, whereas contaminant-tolerant photocatalysts could reduce pretreatment requirements and significantly lower the overall cost of green hydrogen production. Therefore, the selection of appropriate water treatment technologies should consider contaminant composition, treatment efficiency, energy consumption, capital cost, and compatibility with the photocatalytic process.^63,64^

### 8.2. Removal of suspended solids

Suspended solids, including clay particles, silt, colloids, and other particulate matter, reduce light penetration and increase turbidity, thereby limiting the amount of solar radiation reaching photocatalyst surfaces. Particles may also physically cover catalyst active sites or promote fouling of reactor components. Conventional treatment methods such as sedimentation, coagulation–flocculation, rapid sand filtration, and membrane filtration are widely employed to remove suspended solids before advanced water treatment. Ultrafiltration and microfiltration membranes can effectively eliminate particles larger than approximately 0.01–0.1 µm while maintaining relatively low energy consumption.^65^

### 8.3. Removal of heavy metals

Heavy metals such as Pb^2+^, Cd^2+^, Ni^2+^, Cr^3+^/Cr^6+^, Cu^2+^, Fe^3+^, Hg^2+^, and As species are among the most harmful contaminants affecting photocatalytic hydrogen production. These ions can adsorb strongly onto catalyst surfaces, occupy catalytic active sites, alter semiconductor electronic structures, and promote charge-carrier recombination. Heavy metal removal is commonly achieved using chemical precipitation, ion exchange, adsorption, membrane filtration, electrocoagulation, and advanced adsorbents such as activated carbon, zeolites, biochar, metal–organic frameworks (MOFs), and functionalized nanomaterials. Among these methods, adsorption has gained significant attention because of its simplicity, high removal efficiency, and low operating cost. Membrane processes such as nanofiltration and reverse osmosis can also achieve removal efficiencies exceeding 95% for many heavy metals but require higher energy input.^66–68^

### 8.4. Removal of inorganic ions

Natural waters contain high concentrations of chloride, sulfate, nitrate, phosphate, bicarbonate, carbonate, calcium, magnesium, sodium, and potassium ions. Some of these ions can compete with reactants for adsorption sites, scavenge reactive oxygen species, or induce catalyst deactivation through precipitation and scaling. Water softening using lime treatment, ion exchange resins, electrodialysis, nanofiltration, and reverse osmosis are widely used to reduce inorganic ion concentrations. Reverse osmosis is particularly effective for producing high-purity water suitable for hydrogen production but significantly increases capital and operating costs due to its high-pressure requirements.^69^

### 8.5. Removal of natural organic matter

Natural organic matter, including humic acids, fulvic acids, proteins, and polysaccharides, is widely present in surface waters and wastewater. NOM strongly adsorbs onto photocatalyst surfaces, blocks active sites, absorbs incident light, scavenges reactive radicals, and promotes membrane fouling. Conventional coagulation using aluminum or iron salts remains one of the most economical methods for NOM removal. Activated carbon adsorption, biological activated carbon, ozonation, membrane filtration, and advanced oxidation processes further improve NOM removal efficiency. Activated carbon remains one of the most widely adopted technologies because of its high adsorption capacity and operational simplicity.

### 8.6. Removal of microorganisms

Microbial contamination contributes to biofilm formation, catalyst fouling, and degradation of reactor performance during long-term operation. Disinfection technologies include chlorination, ozonation, ultraviolet irradiation, membrane filtration, and advanced oxidation processes. UV disinfection is particularly attractive because it introduces no residual chemicals that might interfere with photocatalytic hydrogen production.^70^

### 8.7. Desalination of seawater

Because seawater represents the largest available water resource, its utilization for hydrogen production has attracted increasing interest. However, high salinity, chloride ions, magnesium, calcium, sulfate, and biological contaminants significantly reduce photocatalyst stability and hydrogen evolution performance. Reverse osmosis remains the dominant desalination technology worldwide due to its high salt rejection (>99%), although it is associated with considerable energy consumption. Emerging technologies, including membrane distillation, capacitive deionization, forward osmosis, and solar-driven desalination, are being investigated to reduce energy demand and improve sustainability.^71^

### 8.8. Advanced oxidation processes (AOPs)

Advanced oxidation processes (AOPs) generate highly reactive hydroxyl radicals (˙OH) and sulfate radicals (SO_4_˙^−^) capable of degrading persistent organic contaminants before photocatalytic hydrogen production. Technologies such as ozone/UV, UV/H_2_O_2_, Fenton oxidation, photo-Fenton reactions, and persulfate activation effectively remove refractory organic compounds that cannot be eliminated using conventional treatment methods. Although AOPs improve water quality substantially, they also increase energy consumption and operational costs, requiring careful economic evaluation for large-scale hydrogen production.^72^

## 9. Future perspectives and research directions for water-quality-aware photocatalytic hydrogen production

Although photocatalytic water splitting has achieved remarkable progress in terms of catalyst development, reaction mechanisms, and hydrogen evolution efficiency, its transition from laboratory-scale demonstrations to practical hydrogen production remains limited by challenges associated with catalyst durability, realistic water environments, scalability, and economic feasibility (Fig. 7). Future research should move beyond the conventional strategy of optimizing only initial hydrogen evolution rates and instead focus on developing stable, efficient, and contaminant-tolerant photocatalytic systems capable of operating under realistic water conditions. As Domen and co-workers^37^ emphasized, the practical implementation of photocatalytic water splitting requires simultaneous improvement of solar-to-hydrogen efficiency, catalyst stability, and large-scale reactor performance rather than focusing on individual performance parameters alone.^22^

**Fig. 7:**
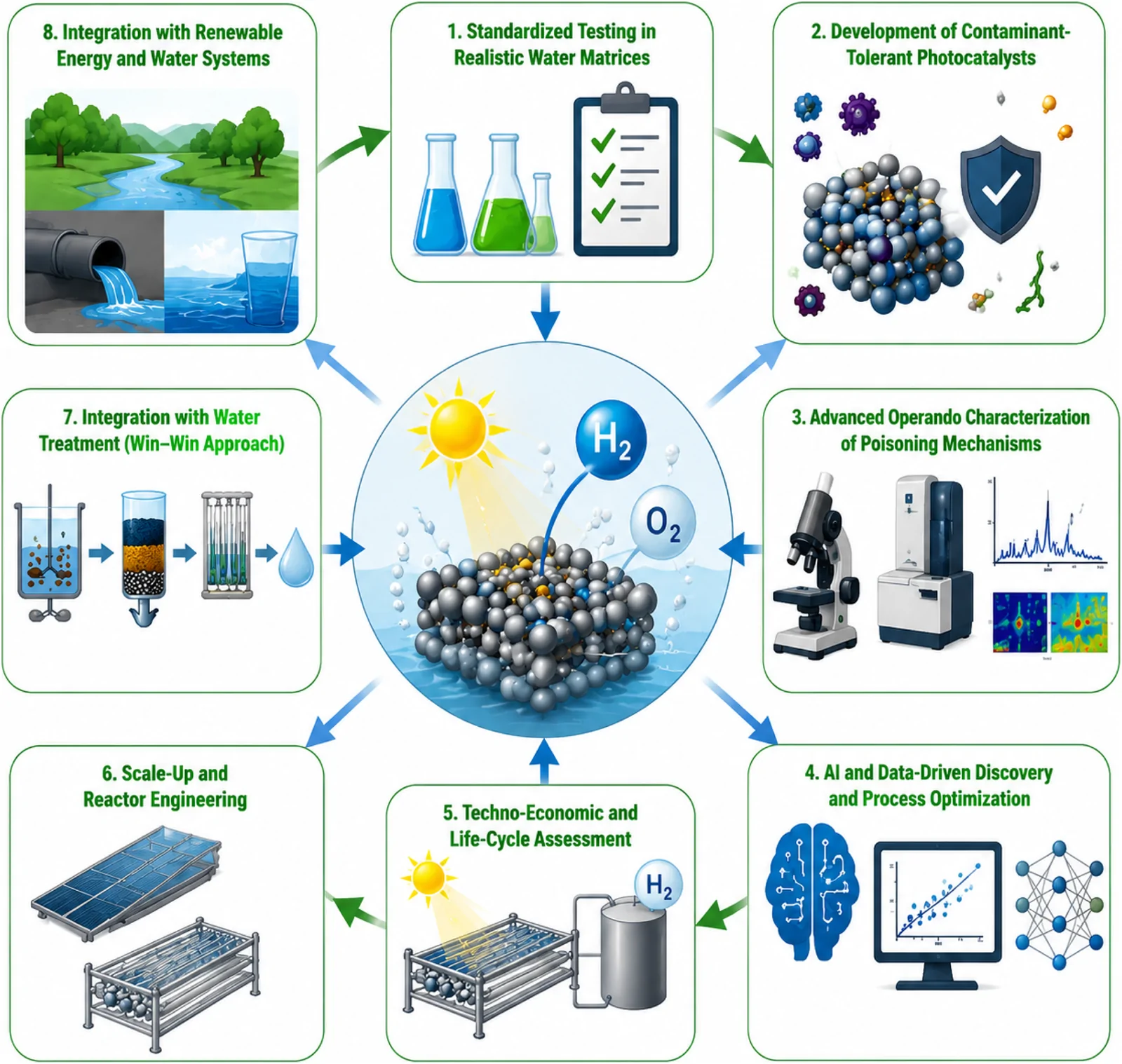
Future research roadmap for water-quality-aware photocatalytic hydrogen production.

A major future direction is the establishment of standardized evaluation protocols using realistic water matrices. Currently, most photocatalytic hydrogen studies employ ultrapure or deionized water to avoid interference from dissolved ions, organic matter, and suspended solids. Although this approach provides information about intrinsic catalyst activity, it does not represent the complex chemical environment encountered in natural waters, wastewater, or seawater. Future studies should systematically evaluate photocatalysts in representative water matrices containing realistic concentrations of heavy metals, inorganic ions, natural organic matter, and particulate pollutants. Nishioka *et al.*^1^ highlighted that future photocatalytic water-splitting research must include realistic operational conditions and long-term stability measurements to bridge the gap between academic studies and industrial applications. Establishing internationally accepted testing protocols, including standardized contaminant concentrations, reaction duration, and reporting methods, will enable meaningful comparison between different photocatalytic systems.

The development of contaminant-resistant photocatalysts represents another critical research priority. Conventional catalyst design has primarily focused on improving light absorption, charge separation, and surface reaction kinetics; however, future materials should also be engineered to resist surface poisoning and structural degradation. Strategies including surface protection layers, core–shell architectures, defect engineering, oxygen vacancy optimization, hydrophobic modification, and robust cocatalyst design may provide improved resistance against heavy-metal adsorption, organic fouling, and salt accumulation. Osterloh^51^ emphasized that catalyst stability and resistance to environmental degradation are essential factors determining the practical success of photocatalytic systems, particularly when moving from controlled laboratory conditions to complex aqueous environments (Osterloh, 2008). In addition, multifunctional photocatalysts capable of simultaneously producing hydrogen and removing contaminants from wastewater could provide additional environmental and economic benefits. Advanced characterization techniques will also play an increasingly important role in understanding and controlling catalyst poisoning mechanisms. Conventional characterization methods performed before and after reactions often fail to capture dynamic interactions between contaminants and catalyst surfaces. Future investigations should integrate *operando* techniques, including synchrotron-based X-ray absorption spectroscopy (XAS), ambient-pressure X-ray photoelectron spectroscopy (AP-XPS), *in situ* infrared spectroscopy, Raman spectroscopy, and transient spectroscopic methods, to monitor surface reconstruction, contaminant adsorption, and charge-transfer processes during photocatalytic operation. Kranz and Wächtler^35^ emphasized that combining advanced spectroscopic techniques with photocatalytic measurements provides essential insights into the relationship between catalyst structure, charge dynamics, and catalytic performance.

Integration with renewable energy and water treatment systems offers an effective strategy to reduce the contaminant load in feedwater before photocatalytic hydrogen production. Renewable-powered pretreatment technologies, including membrane filtration, adsorption, solar-driven desalination, and advanced oxidation processes, can remove heavy metals, natural organic matter, suspended solids, and excess inorganic ions that are responsible for catalyst poisoning. By lowering the concentration of these contaminants, the integrated system minimizes active-site blocking, surface fouling, and charge-carrier recombination, thereby improving catalyst durability, maintaining stable hydrogen production, and reducing the need for extensive chemical pretreatment. Consequently, coupling renewable energy with water treatment and photocatalytic hydrogen production enhances both the technical performance and economic sustainability of green hydrogen systems.^73^

Artificial intelligence (AI) and machine learning (ML) are expected to accelerate the discovery of next-generation photocatalysts by establishing relationships between material properties, water chemistry, and catalytic performance. Machine-learning models can predict catalyst activity, contaminant adsorption behavior, degradation pathways, and optimal synthesis conditions using large experimental and computational datasets. Butler *et al.*^60^ demonstrated that machine learning has the potential to transform materials discovery by rapidly identifying promising materials and reducing dependence on conventional trial-and-error approaches. In photocatalytic hydrogen production, AI-driven approaches could predict catalyst poisoning under different water compositions, optimize reactor operation, and support the development of digital twins for real-time monitoring of catalyst performance. Such intelligent systems could enable adaptive hydrogen production processes that respond dynamically to variations in feedwater quality.

Economic analysis and sustainability assessment should become integral components of future photocatalytic hydrogen research. Although highly active photocatalysts are continuously being developed, their commercial viability depends on factors including catalyst lifetime, regeneration ability, water pretreatment requirements, reactor design, and operational costs. Maurya *et al.*^36^ demonstrated through techno-economic modelling that catalyst durability and system efficiency strongly influence the levelized cost of hydrogen (LCOH), indicating that improvements in stability may provide greater economic benefits than simply increasing initial hydrogen evolution rates. Therefore, future studies should combine techno-economic analysis with life-cycle assessment to evaluate water consumption, energy demand, carbon emissions, and environmental impacts throughout the entire hydrogen production process.

Finally, large-scale implementation requires significant advances in reactor engineering and integration with renewable energy infrastructure. Most reported photocatalytic studies are conducted at laboratory scale under optimized illumination conditions, whereas industrial applications require large-area reactors, efficient photon utilization, catalyst recovery systems, and continuous operation using realistic water sources. Wang and Domen^37^ emphasized that scalable reactor design, efficient solar utilization, and long-term operational stability remain key challenges for practical photocatalytic hydrogen production (Wang and Domen, 2020). Future systems should integrate photocatalytic hydrogen production with wastewater treatment facilities, desalination processes, and renewable energy networks to maximize resource utilization and minimize environmental impacts.

Overall, the future of photocatalytic hydrogen production will depend on a paradigm shift from developing highly active catalysts under ideal laboratory conditions toward designing water-quality-aware, economically viable, and environmentally sustainable photocatalytic systems. Addressing contaminant-induced catalyst poisoning through advanced materials engineering, real-time characterization, AI-assisted optimization, and integrated water-energy strategies will be essential for transforming photocatalytic water splitting into a practical technology for the global hydrogen economy.

Future research should progress through clearly defined and measurable milestones rather than focusing solely on incremental improvements in hydrogen evolution rates. In the short term, internationally standardized protocols should be established for evaluating photocatalysts under representative water matrices containing defined concentrations of inorganic ions, heavy metals, natural organic matter, and suspended solids. Medium-term efforts should focus on developing contaminant-tolerant photocatalysts capable of maintaining at least 90% of their initial hydrogen production activity after more than 100 h of operation in realistic water environments while simultaneously reducing pretreatment energy requirements. Long-term objectives should include pilot-scale demonstrations operating continuously for more than 1000 h, integration of AI-assisted catalyst design and digital twin technologies, and comprehensive techno-economic assessments targeting reductions in the levelized cost of hydrogen (LCOH). Achieving these milestones will accelerate the transition of photocatalytic hydrogen production from laboratory research to commercially viable green hydrogen technologies.

## 10. Conclusion

Photocatalytic water splitting represents a promising pathway for sustainable hydrogen production by directly converting solar energy into chemical fuel using water as the primary resource. Despite significant progress in semiconductor development, cocatalyst engineering, heterojunction construction, and charge-transfer optimization, the practical implementation of photocatalytic hydrogen production remains limited by challenges associated with efficiency, stability, scalability, and economic feasibility. Among these challenges, the role of water quality has received considerably less attention despite its critical influence on catalyst performance and long-term operation. This review highlights that water should not be considered merely as a reactant but as a complex chemical environment capable of controlling photocatalyst activity, durability, and overall process economics. The presence of contaminants in natural waters and wastewater, including heavy metal ions, inorganic anions, natural organic matter, suspended solids, and emerging pollutants, can significantly influence photocatalytic hydrogen production through multiple poisoning pathways. These include active-site blocking, competitive adsorption, charge-carrier recombination, surface fouling, photocorrosion, crystal structure degradation, cocatalyst poisoning, and radical scavenging. Such interactions can decrease hydrogen evolution rates, accelerate catalyst deactivation, and shorten operational lifetime. Therefore, catalyst evaluation based exclusively on ultrapure water conditions may overestimate practical performance and does not accurately represent real-world operating environments.

Advanced characterization techniques, including *operando* spectroscopy, synchrotron-based X-ray absorption spectroscopy, Kelvin probe force microscopy, electrochemical impedance spectroscopy, transient optical spectroscopy, and computational approaches such as density functional theory and molecular dynamics simulations, provide valuable opportunities to understand contaminant–catalyst interactions at molecular and atomic levels. Integrating these approaches will enable identification of active degradation pathways and facilitate the rational design of photocatalysts with improved resistance against complex water chemistries.

Water pretreatment strategies, including filtration, adsorption, ion exchange, membrane separation, desalination, and advanced oxidation processes, can effectively reduce contaminant concentrations and improve catalyst stability. However, extensive purification increases energy consumption and operational costs, creating an important trade-off between water quality improvement and hydrogen production economics. Consequently, future technologies should focus not only on removing contaminants but also on developing water-quality-tolerant photocatalysts capable of maintaining high activity under realistic conditions. The economic analysis of photocatalytic hydrogen production demonstrates that catalyst lifetime, water treatment requirements, and operational stability are critical factors determining the levelized cost of hydrogen. Reducing dependence on expensive ultrapure water and minimizing pretreatment requirements through robust catalyst design could significantly improve the competitiveness of photocatalytic hydrogen production. Therefore, techno-economic assessment and life-cycle analysis should be integrated into catalyst development at an early stage rather than considered only after achieving high laboratory-scale activity. Emerging technologies, particularly artificial intelligence, machine learning, and digital twin approaches, provide new opportunities to accelerate the discovery of contaminant-resistant photocatalysts, predict poisoning behavior, optimize operating conditions, and enable real-time management of photocatalytic systems. Future research should combine experimental databases, computational modelling, advanced characterization, and AI-driven prediction tools to establish intelligent photocatalytic platforms capable of adapting to changing water conditions.

## Author contributions

Afsar Khan: writing original draft; Munevver Tuna Genc, conceptualization, review and editing; Imren Hatay Patir: conceptualization, literature review; Mustafa Ersoz edited the final version of paper.

## Conflicts of interest

The authors declare that they have no known competing financial interests or personal relationships that could have appeared to influence the work reported in this paper.

## Data Availability

The data that support the findings of this study are available from the corresponding author upon reasonable request. All relevant data generated or analyzed during this study are included in the article.
